# Effect of Storage Time and Temperature on Phenolic Compounds of Soybean (*Glycine max* L.) Flour

**DOI:** 10.3390/molecules23092269

**Published:** 2018-09-05

**Authors:** Mayakrishnan Prabakaran, Ji-Hee Lee, Ateeque Ahmad, Seung-Hyun Kim, Koan-Sik Woo, Mi-Jung Kim, Ill-Min Chung

**Affiliations:** 1 Department of Crop Science, College of Sanghuh Life Science, Konkuk University, 120 Neungdong-ro, Gwangjin-gu, Seoul 05029, Korea; prabakarannitt@gmail.com (M.P.); j1225@konkuk.ac.kr (J.-H.L.); kshkim@konkuk.ac.kr (S.-H.K.); 2 Process Chemistry and Technology Department, CSIR-Central Institute of Medicinal and Aromatic Plants, Lucknow 226015, India; ateeque97@gmail.com; 3 Department of Central Area Crop Science, National Institute of Crop Science, Rural Development Administration, Suwon 16429, Korea; wooks@korea.kr; 4 Department of Central Area Crop Science, National Institute of Crop Science, RDA, Suwon 16613, Korea; tyche@korea.kr

**Keywords:** soybean flour, phenolic acids, isoflavones, LC/MS-MS

## Abstract

The phenolic compounds (PC) of soybeans (*Glycine max* (L.) Merrill) varies mainly based on factors like genetics, the environment, and also the food processing techniques used. The effect of storage time and temperature on the phenolic acids and isoflavones composition of raw soybean flour (RWSF) and roasted soybean flour (RSF) were analyzed using liquid chromatography-tandem mass spectrometry (LC-MS/MS). Based on the analysis results, 56 PC and free amino acids were detected in the RWSF and RSF. The total phenolic content (TPC) was 301.59 µg/g in the control RWSF and 257.47 µg/g in the control RSF. In the analysis, eight types of phenolic acids and one flavonoid group belonging to the isoflavone group were detected. When comparing storage conditions of RWSF, 24 and 48 weeks of storage showed higher concentration of phenolic acids. In RSF, the percentage of total acetyl glucosides was high, but the outcome was reversed after 2 weeks. This study had identified that the composition of PC in RWSF and RSF were affected when the storage temperature increased and the storage time lengthened.

## 1. Introduction

Soybeans have been an important food source in Asian countries for centuries. Soybeans were traditionally consumed in two different forms, fermented and nonfermented. Soybean sprouts, tofu, and soymilk are included in the non-fermented soy foods, whereas soy sauce, soybean paste, natto, and tempeh are fermented soy foods. In the USA, soybeans are mainly considered as rich protein sources that are usually utilized as ingredients in various food preparations [1]. Soybeans are also well known as a good source of various nutrients. Generally, soybeans consist of protein, fat, soluble carbohydrates such as raffinose and stachyose, and insoluble carbohydrate such as dietary fiber [2,3]. Soybeans also contain a variety of nonnutritive components called bioactive phytochemicals, such as isoflavones, phenolic acids, phytosterols, and saponins [4]. Because of these kinds of phytochemicals in soybeans, they are considered to have beneficial effects on human health. Furthermore, regular consumption of soybean or soy products helps reduce or prevent some chronic diseases such as cancer, cardiovascular disease, and osteoporosis. In particular, the occurrence of breast and prostate cancer were ideally reduced by the soybean isoflavones [5,6], Soybean compounds also reduce rapid bone loss, and decrease bone resorption in menopausal women [7]. Additionally, the intake of soy proteins or isoflavones lowers LDL-cholesterol levels and serum total cholesterol [8,9]. Thus, much of the growth of the soy foods market is attributed to the increased interest of consumers in health issues and their awareness of soybeans as a healthy beneficial food ingredient [10].

The PC composition of soybeans varies based on genetic and ecological factors, such as cultivar and cultivation location [11,12]. Additionally, the various food processing techniques used can also affect the PC composition of soy foods [13,14]. Many studies regarding the effect of processing on phenolic compounds, especially isoflavones in soy foods, have been performed. Isoflavones found in soybeans are composed of β-glucosides, acetyl-β-glucosides, malonyl-β-glucosides and aglycones. Among them, malonyl-β-glucoside is the most prominent in raw soybeans, and the least is aglycone [15]. Soybean products made with mild heating treatment were reported to have similar isoflavone composition to raw soybeans, with the highest proportion being malonyl-β-glucosides. On the other hand, comparatively stronger heating treatment when making soy milk or cooked soybeans caused a decrease in malonyl-β-glucosides. Furthermore, fermented soy foods, such as natto and chunggukjang, have a higher proportion of aglycones as compared to raw soybeans [16]. This occurs because during fermentation, the β-glucosidase generated from microorganisms hydrolyzes and converts β-glucosides into aglycones [17,18]. In the case of roasting, frying, and baking, the amounts of acetyl-β-glucosides increases because of decarboxylation of malonyl derivatives [19]. Similarly, in the production of kinako (RSF used in Japanese cuisine), the proportion of acetyl-β-glucosides increased, whereas the amount of malonyl-β-glucosides decreased [16].

Among many kinds of foods that are made from soybeans, soybean flour is one of the widely used preparations that are easily accessible to consumers. Soybean flour is commonly produced by finely grinding full-fat soybeans after removing the hull, and more than 97% of the flour must pass through a 100 standard mesh to be called soybean flour [1]. The use of soybean flour is diverse, and a common example is its use as a protein source in industrial fermentations [20].

During soy processing and cooking change in contents of phenolic acids or isoflavones were observed [21,22,23,24]. However, there are few studies regarding the variation in PC depending on different storage conditions and durations. Understanding the changes in PC in RWSF and RSF during storage will advance our knowledge regarding the extent the PC, especially isoflavones, can be retained during storage. It will also provide a basis for providing better storage conditions and increasing shelf life of soybean flour that can retain the beneficial bioactive compounds. Therefore, during this study, the changes in composition and content of PC in RWSF and RSF during storage for one year at three different temperatures were analyzed using LC-MS/MS. The content and composition of 56 selected PC and free amino acids were monitored to determine the effects of the conditions and storage time on the profile of PC in soybean flour.

## 2. Results and Discussion

### 2.1. Profiles of Phenolic Compounds (PC) in Raw Soybean Flour (RWSF) and Roasted Soybean Flour (RSF)

**Figure 1:**
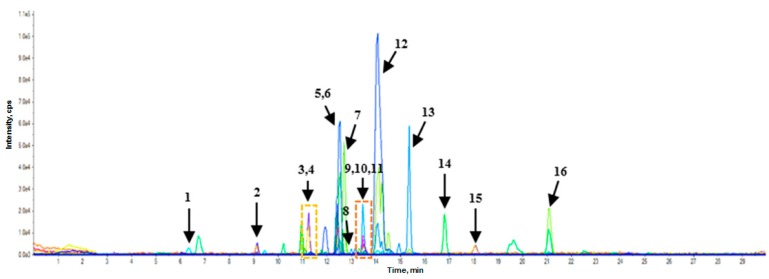
Multiple reactive monitoring (MRM) ion chromatogram of control raw soybean flour (RWSF). (**1**) l-phenylalanine; (**2**) protocatechuic acid; (**3**) daidzin; (**4**) *p*-hydroxybenzoic acid; (**5**) malonylglycitin; (**6**) malonyldaidzin; (**7**) genistin; (**8**) *β*-resorcylic acid; (**9**) acetyldaidzin; (**10**) acetylglycitin; (**11**) *p*-coumaric acid; (**12**) malonylgenistin; (**13**) acetylgenistin; (**14**) daidzein; (**15**) salicylic acid; (**16**) genistein.

**Figure 2:**
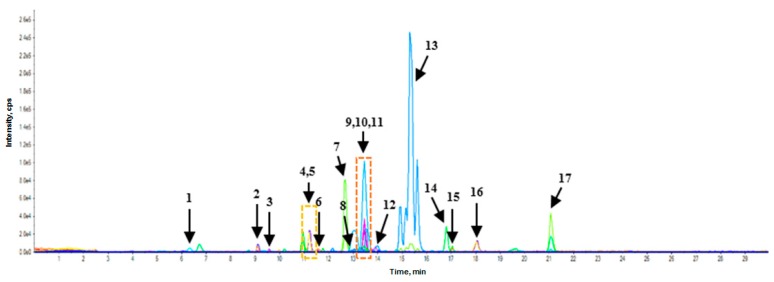
MRM ion chromatogram of control roasted soybean flour (RSF). (**1**) l-phenylalanine; (**2**) protocatechuic acid; (**3**) chlorogenic acid; (**4**) daidzin; (**5**) *p*-hydroxybenzoic acid; (**6**) syringic acid; (**7**) genistin; (**8**) *β*-resorcylic acid; (**9**) acetyldaidzin; (**10**) acetylglycitin; (**11**) *p*-coumaric acid; (**12**) ferulic acid; (**13**) acetylgenistin; (**14**) daidzein; (**15**) glycitein; (**16**) salicylic acid; (**17**) genistein.

Through the analysis of 56 selected PC and amino acids, 20 PC and one amino acid were detected in the RWSF and RSF. Figure 1 and Figure 2 show the multiple reactive monitoring (MRM) ion chromatogram of control RWSF and RSF.

The composition of PC in RWSF and RSF are shown in Table 1, Table 2 and Table 3.

**Figure 3:**
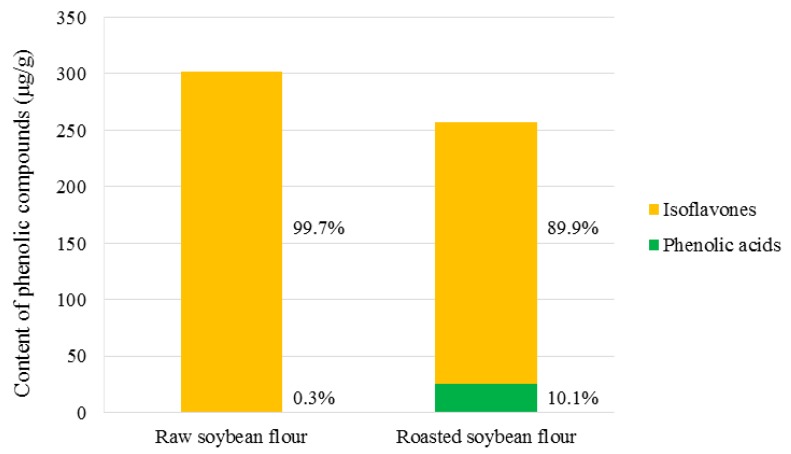
Composition of the PC in RWSF and RSF.

The content of TPC was 301.59 µg/g in control RWSF and 257.47 µg/g in control and RSF. Among PC, eight types of phenolic acid, including protocatechuic acid, *p*-hydroxybenzoic acid, syringic acid, salicylic acid, *β*-resorcylic acid, *p*-coumaric acid, chlorogenic acid, and ferulic acid, were found in the soybean flour samples. In RWSF from the control flour to flour after 1 year of storage, syringic acid and chlorogenic acid were ND (not detected). Ferulic acid was below the limit of detection (LOD) or not detected, except in the RWSF stored at HT for 48 weeks. Among the flavonoid groups, compounds that belong to the isoflavone group were only detected in the RWSF and RSF samples. l-phenylalanine, a precursor of PC, was detected in both RWSF and RSF samples. The content of isoflavones was higher than that of phenolic acids in both RWSF and RSF. In the RWSF, the isoflavone group accounted for 99.7% of the TPC and phenolic acids accounted for 0.3%. Similar to RWSF, in the isoflavone group in the RSF, TPC represented 89.9% and phenolic acids 10.1% (Figure 3). 

The content of TPC was higher in RWSF than in RSF. This was because roasting caused a loss in total isoflavones. A previous study [14] reported loss in total isoflavones during roasting of soybeans and the loss occurred more during roasting because of the HT treatment.

**Figure 4:**
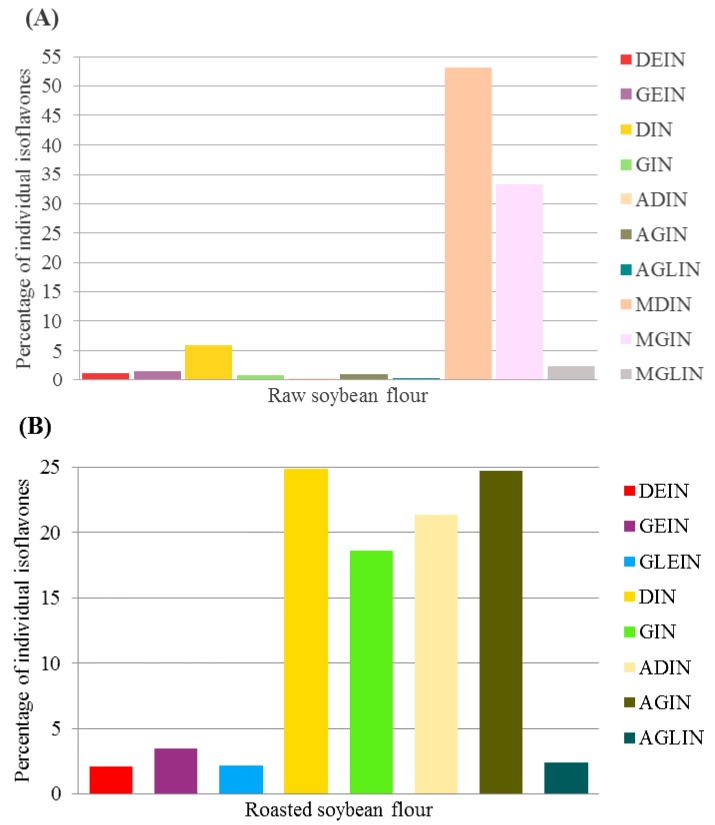
Composition of isoflavones in (**A**) RWSF and (**B**) RSF. The percentage of individual isoflavones indicates the ratio of each isoflavone content of aglycone equivalents over total isoflavone content of aglycone equivalents. DEIN: daidzein, GEIN: genistein, GLEIN: glycitein, DIN: daidzin, GIN: genistin, ADIN: acetyldaidzin, AGIN: acetylgenistin, AGLIN: acetylglycitin, MDIN: malonyldaidzin, MGIN: malonylgenistin, MGLIN: malonylglycitin.

Isoflavones are primarily found in the form of flavonoids in soybeans and can be divided into four groups, including acetyl glucosides, aglycones, malonyl glucosides and glucosides, according to the glucoside part. Among the 12 isoflavones identified, the proportion of malonyl daidzin was the highest (53.2% of the total isoflavones) in the RWSF. Conversely, daidzin was the most abundant (24.9% of the total isoflavones) in the RSF, followed by acetyl genistin (24.7% of the total isoflavones) and acetyl daidzin (21.4% of the total isoflavones) (Figure 4).

**Figure 5:**
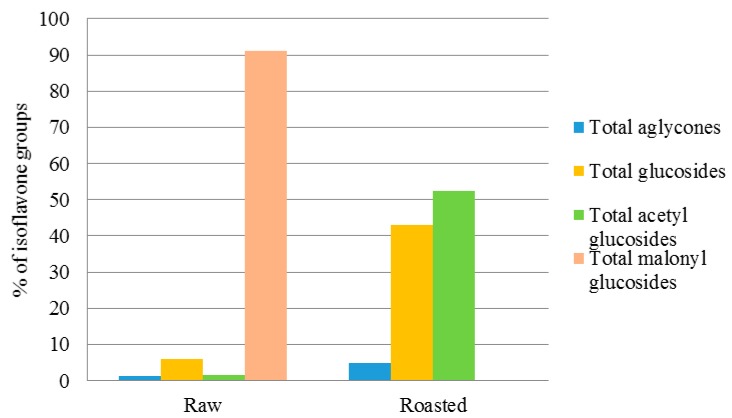
Comparison of four isoflavone groups in RSF and RWSF.

Among the four isoflavone groups, the malonyl glucosides accounted for 91.2% of the total isoflavones in the control RWSF. The glucosides group was the next most abundant isoflavone representing 5.9% of the total isoflavones. Of the glucosides group, however, the content of glycitin in the control RWSF was below the LOD. Aglycone and acetyl glucoside groups were responsible for 1.4 and 1.5%, respectively. In the control RWSF, malonyl glucosides were not detected or below the LOD. The acetyl glucosides group was the most abundant isoflavone in the RSF (52.3% of the total isoflavones), followed by glucosides (42.9% of the total isoflavones) and alycones (4.8% of the total isoflavones) (Figure 5).

**Figure 6:**
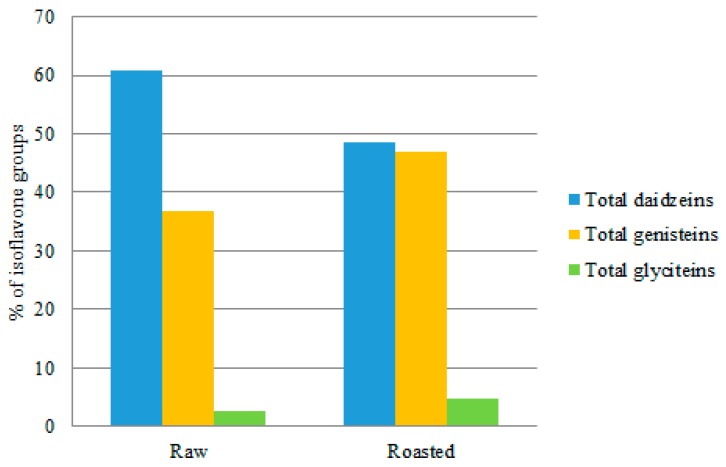
Comparison of three isoflavone types in RWSF and RSF.

The most abundant isoflavone group in the RWSF, malonyl glucosides, decreased, whereas acetyl glucosides increased in the RSF. This result corresponds with the previous study [16]. The reduction of malonyl glucosides in RSF flour compared to RWSF was speculated to be a consequence of decarboxylation of malonyl glucosides by the roasting process. As a result, the proportion of acetyl glucosides and glucosides in the RSF was much higher than in the RWSF. When isoflavones were categorized into three types, total daidzeins (i.e., daidzein, daidzin, acetyldaidzin, malonyldaidzin) accounted for the highest proportion in both RWSF and RSF (60.7 and 48.5% of the total isoflavones, respectively) (Figure 6).

The proportion of total genisteins was the second highest (36.8 and 46.8% of the total isoflavones, respectively) and the proportion of total glyciteins was the least in both RWSF and RSF. The RSF exhibited a decrease in the percentage of total daidzeins compared to that of RWSF. According to earlier studies, depending on the experimental conditions genistin and daidzin derivatives had different stability. It was reported that during heating daidzin produced more stability than genistin or glycitin during heating [23]. However, according to another study [25], isoflavone groups loss were observed more in daidzin under elevated temperature. It was also reported if over-dried at 100 °C the malonyldaidzin of soybeans had a steeper slope of decrease than malonylgenistin indicating heat sensitive nature of malonyldaidzin than malonylgenistin [14].

### 2.2. Changes in Phenolic Acids and Isoflavones Based on the Storage Time and Temperature

The changes of composition of PC in RWSF and RSF stored under each storage time are shown in Table 1, Table 2 and Table 3. Table 4 shows the effects and interactions of soybean flour type, storage time, and temperature on the composition of the PC in soybean flour samples. The contents of TPC and total isoflavones, and the sum of phenolic acids and isoflavones were significantly (*p* < 0.0001) affected by each soybean flour type, storage temperature, and time. However, the effects that each factor had on the content of TPC and isoflavones were not different.

Also, the interactions of three factors on the contents of sum of PC were equally significant (*p* < 0.0001).

**Figure 7:**
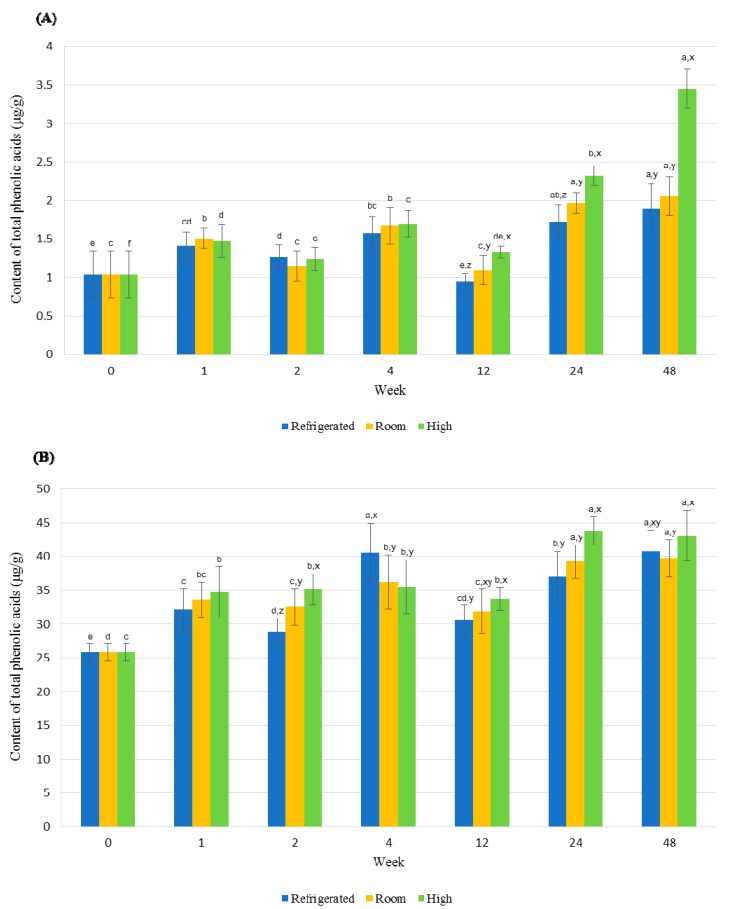
Comparison of the total phenolic acids in (**A**) RWSF and (**B**) RSF according to storage time and temperature. The blue, yellow, and green-colored bars indicate 4, 20 and 45 °C temperature, respectively. ^a–f^ Values with different statistically different superscripts depending on storage time (control, after 1, 2, 4, 12, 24, and 48 weeks) (*p* < 0.05). ^x–z^ Values with different statistically different superscripts between storage temperature (RFT, RT, and HT) (*p* < 0.05).

The changes in content of total phenolic acids depending on storage time and temperature are shown in Figure 7.

At the control stage (represented as 0 in Figure 7), the total phenolic acids content was 1.04 µg/g in RWSF and 25.88 µg/g in RSF. Significant differences among different storage time were observed after 12, 24, and 48 weeks of storage of RWSF. Among the three storage time, the RWSF stored at the 45 °C condition had a higher TPC content then that of RWSF stored at a 4 °C.

No changes were observed in the total phenolic acids for different storage parameters (4, 20 and 45 °C). However, from 12 to 48 weeks of storage, an increase in the total phenolic acids content was seen. The RWSF for 48 weeks had the highest total phenolic acids content in all three storage time. For RSF, significant differences in content of phenolic acids were found after 2 weeks of storage. The total phenolic acids content in RSF stored at HT was the highest, except at 4 weeks of storage. The changes in total phenolic acids content in RSF also showed no constant tendency in all three storage temperature regarding storage time. However, the RSF stored from 12 to 48 weeks increased in all three storage temperatures. Among storage time, 48 weeks showed the highest total phenolic acids content in the RSF of all three storage temperature, but the contents of total phenolic acids in the RSF stored at RT and HT had no significant difference between 24 and 48 weeks.

The results had identified that the total phenolic acids content of both RWSF and RSF were affected by the storage temperature and time. According to the increase in storage temperature and time, the total phenolic acids content in RWSF and RSF also increased when compared to the control stage. Studies on changes in isoflavones in soybeans and soy foods according to storage temperature and food processing are well established, but the changes in phenolic acids content and composition in soybeans according to storage temperature are limited. In previous studies, the contents of PC in soybeans decreased according to cooking or processing. When soybeans were cooked by electric rice cooker, the PC in soybean decreased by 12 and 8%, respectively, exhibiting a significant difference (*p* < 0.05) compared to the soybeans before cooking [26]. Significant decrease (*p* < 0.05) in TPC was shown on the legumes that are steamed or boiled [27]. These significant losses of phenolics could be due to the breaking down of PC during thermal process or by leaching by cooking water. However, processing does not always cause a decrease in total phenolics. In case of yellow soybeans, the process of regular boiling or steaming caused significant (*p* < 0.05) reductin in TPC, whereas significant (*p* < 0.05) increases was observed during pressure steaming [28]. It was also reported that the steam cooking of broccoli caused an increase in the content of TPC, contrary to the influence of boiling [29]. In case of spinach, the amount of total phenolics decreased after storage at different temperatures (4 °C or −18 °C), whereas the individual phenolic acids content in spinach increased. Specifically, spinach when boiled after 1 month of storage showed a significant (*p* < 0.05) increase in *o*-coumaric acid, *p*-coumaric acid, and ferulic acid [30]. The present study of the RWSF and RSF were packed in sealed polyethylene film bags during storage, so unlike cooking of soybeans there was no leaching or loss of PC through cooking waters. Therefore, these result shows that the increase in the amount of phenolic acids in both RWSF and RSF can be explained continuation of improved release of phenolic acids from the soybean matrix or the degradation of complex polymerized phenolic structures into simple phenolic structures as the storage temperature becomes higher and the storage time longer.

**Figure 8:**
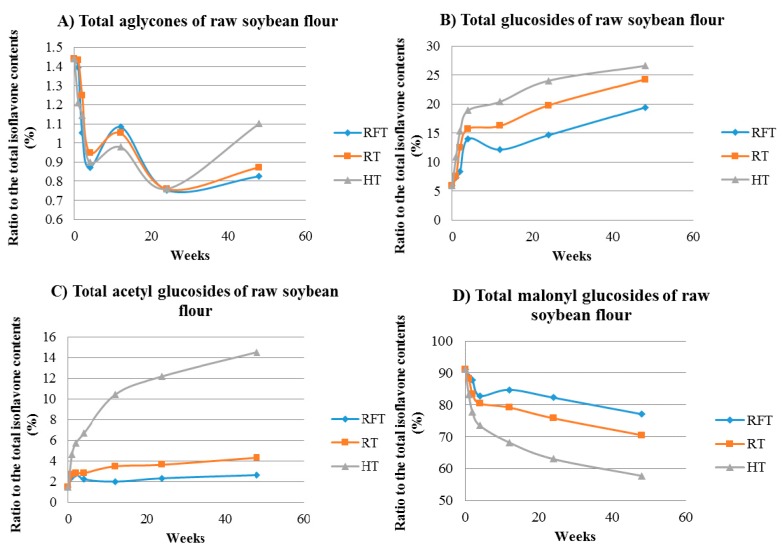

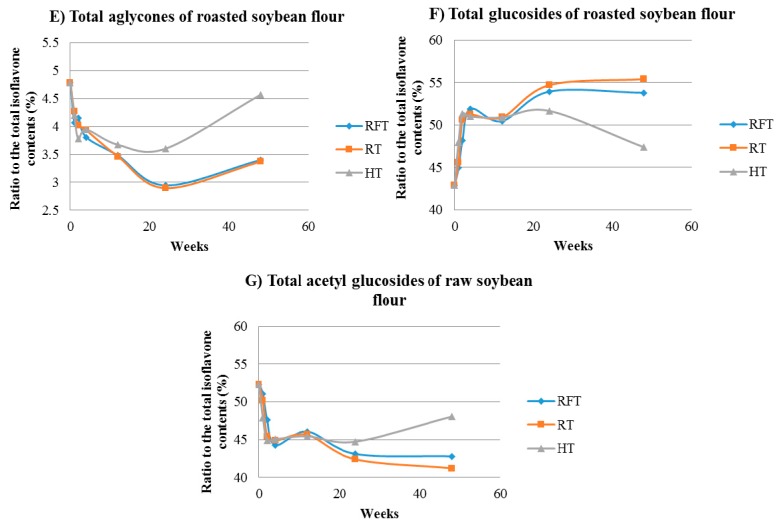
Changes of the isoflavone groups according to the storage time (control, 1, 2, 4, 12, 24, and 48 weeks) and temperature. RFT: Refrigerated temperature (4 °C); RT: Room temperature (20 °C); HT: High temperature (45 °C). Isoflavone content of each storage time indicates the ratio of the sum of each isoflavone group over the sum of total isoflavones.

The changes in the proportion of each isoflavone group depending on the storage condition and time are shown in Figure 8.

The percentage of the each isoflavone group (i.e., aglycones, glucosides, acetyl glucosides, and malonyl glucosides) over the total isoflavones was used to observe the changes in between isoflavone group composition. This was similar to the total isoflavone groups in RWSF stored at three different temperatures (RFT, RT, and HT) for 1 year. In RWSF, the percentage of total malonyl glucosides over the sum of isoflavones was highest at the control storage, accounting for 91.2% of the total isoflavones, but decreased as the storage time became longer. The degree of reduction in malonyl glucosides increased as the storage temperature increased. The percentage of total malonyl glucosides in RWSF stored for 48 weeks at 4 °C decreased by 77.1%, 70.5% at 20 °C, and 57.8% at 40 °C. Conversely, the % of total glucosides increased following the storage time. The RWSF stored at 40 °C showed a rapid decline in the % total malonyl glucosides but an increase in the % total glucosides and acetyl glucosides. It is thought that the degradation of malonyl glucosides was continued as the storage time increased, and it was facilitated when the storage temperature was high. In the RSF, the percentage of total acetyl glucosides accounted for the highest proportion followed by glucosides at the control stage. However, the percent contribution of acetyl glucosides and glucosides to the total amount of isoflavones was reversed after 2 weeks of storage in all three storage time. The % of the total glucosides became higher than that of total acetyl glucosides from 2 weeks, and it continued throughout storage. This tendency shows that the storage time affects the number and distribution among isoflavone groups.

The isoflavones are the abundant PC in soybeans, whereas the malonyl glucosides takes up a large portion of isoflavones in RWSF. The effects of various processing or storage on soybeans and soy foods were researched in many studies [31]. In a previous study, the influences of oven-drying and roasting process on isoflavone compositions in soybeans were investigated [14]. The content of total isoflavones in soybeans roasted at 200 °C for 21 min decreased and the oven-drying at 100 °C for 120 min decreased malonyl glucosides and increased with aglycones and glucosides, but the content of total isoflavones in oven-dried soybeans did not showed any significant (*p* < 0.05) difference from that of untreated soybeans. In another study, there was a significant increase after 10 min of roasting at 200 °C in acetyl glucosides and glucosides and after 20 min of roasting a decrease of acetyl glucosides was observed [16]. Changes in isoflavone content and distribution in soybeans and soy foods are affected not only the processing but also by storage. The loss of genistin in soy milk was seen at elevated storage temperature, and also the content of acetyl daidzin in soy milk stored at 80 and 90 °C increased, followed by a slight decrease [32]. Three years of storage of fifteen soybean cultivars at RT (25 °C) decreased the amount of total isoflavones as the period of storage increased [33]. The content of glucosides and aglycones increased over the storage time, whereas malonyl glucosides decreased significantly. Similar results were reported when the malonyl glucosides in soybeans stored at 80 °C decreased along with the formation of acetyl glucosides and glucosides [34].

The changes in the proportion of isoflavone groups in RWSF and RSF over 48 weeks of storage were consistent with the previous studies. The decrease in the proportion of total malonyl glucosides and increase in the proportion of total glucosides and total acetyl glucosides in RWSF during storage at all three temperatures showed that the conversion of malonyl glucosides into glucosides and acetyl glucosides occurred because of the degradation and decarboxylation of the malonyl group. However, the proportion of aglycones did not increase during storage because the temperature of storage was not high enough or the storage time was not long enough for the cleavage of the glycosidic bond in glucosides. In case of RSF, the reversal of the proportion between acetyl glucosides and glucosides occurred. This result shows that the storage time affects the acetyl group. However, in RSF stored at 45 °C, total glucosides decreased and total acetyl glucosides increased from 24 weeks. This was possibly caused by the conversion of glucosides to aglycones, which decreased the proportion of total glucosides and increased the proportion of total acetyl glucosides. However, further study will be needed to determine whether the change in the proportion in soybean flour is temporary or consistent. In the present study, the changes in the proportion of isoflavone groups corresponded with that of previous studies, however, there was an increase in the content of total isoflavones over the storage time.

Despite the distribution of individual glucoside conjugates, the proportion of phenolic acids in soybeans and soy products kept changing depending on the temperature of processing or extraction. It was reported that the total content of extracted isoflavones was constant unless it was under abnormal cooking conditions like burning [22]. On the contrary, it was also reported that the contents of total isoflavones in yellow and black soybeans significantly (*p* < 0.05) increased when pressure steamed [28]. From this study, the pressure steaming process transformed more malonyl glucosides into glucosides, acetyl glucosides, and alycones than did the regular steaming process and caused higher total isoflavones than regular steaming even in raw yellow and black soybeans. Similarly, in the current study, the amount of isoflavones in both RWSF and RSF increased during storage. The exact reason for the increase of isoflavones in soybean flour over the storage duration remains unclear. However, the release of more isoflavones that were combined with other types of structural substances may be a possible reason.

## 3. Materials and Methods

### 3.1. Sample Preparation

#### 3.1.1. Soybean Flour Preparation

The RWSF and RSF were provided by the Rural Development Administration in 2016. The soybean cultivar ‘Saedanbaek’ was used in this study. The RWSF and RSF were produced by a general process. The soybeans are cleaned and slowly roasted using oven at temperature 110–130 °C for 30 min without breaking the cellular structure or releasing oil content. The raw soybean was ground using a grinder to make RWSF. The RSF was made by roasting the raw soybean and then grinding them. The grind soy bean flour’s were then lyophilized at −47 °C to hold down the moisture content which would be effective to prevent the growth of microbes throughout the storage time [13,35].

#### 3.1.2. Packed Soybean Flour Preparation

The RWSF and RSF were packed in polyethylene (PE) film bags 10 × 15 cm (width × length) and 0.05 mm thickness in size. The three sides of polyethylene film bags were sealed with a sealing machine (Lovero Bag Sealer, SK-310; Sambo Tech Corporation, Gyeonggi-do, Korea) prior to packaging to prevent the PE film bag from bursting. Approximately 30 g of the RWSF and RSF was weighed and then packed in the polyethylene film bags. After putting the soybean flour into the PE film bags, they were sealed by a sealing machine. The packaged samples were prepared in triplicate for both temperature and time of storage.

#### 3.1.3. Temperature and Time of the Soybean Flour Storage

Numerous research studies had identified some changes in the soy bean sub products when stored at 40 and 45 °C. So the highest temperature in this study was chosen as 45 °C to compare the content of phenolic acids and isoflavones with rest of the storage temperatures [36,37]. The RWSF and RSF were stored at three different storage temperatures namely RFT (4 °C), RT (20 °C), and HT (45 °C) for 1 year. The RWSF and RSF were collected once before storing and after storing at different intervals (1, 2, 4, 12, 24, and 48 weeks) to analyze the changes of PC according to the temperature and time of storage.

### 3.2. Chemicals

All solvents used for the extraction and analysis of PC were of HPLC grade. The MeOH (methanol) and acetonitrile were purchased from Fisher Scientific Korea (Seoul, Korea). The 0.1 N hydrochloric acid (HCl) standard solution was purchased from Daejung Chemicals & Metals (Gyeonggi-do, Korea). The formic acid was purchased from Junsei Chemical (Tokyo, Japan). The deionized water was obtained from PURELAB Option-Q System (ELGA Lab Water, UK). The 56 standards of selected PC and free amino acids were obtained as follows. The phenolic acid standards, its derivative standards, amino acid standards, flavonoid standards and dimethyl sulfoxide were purchased from Sigma-Aldrich (St. Louis, MO, USA). The anthocyanin standards were purchased from Extrasynthese (Genay, France). The isoflavone standards were purchased from three different companies; Wako Pure Chemical Industries (Osaka, Japan), LC Laboratories (Woburn, MA, USA) and Sigma-Aldrich (St. Louis, MO, USA). The stilbenoid standards were purchased from two different companies; Sigma-Aldrich (St. Louis, MO, USA) and Cayman Chemical (Ann Arbor, MI, USA).

### 3.3. Extraction of PC

The extraction of PC from RWSF and RSF was performed using the acidic extraction method [38]. A 1 g sample of soybean flour was weighed, added to 10 mL of acetonitrile and 2 mL of 0.1 N HCl, and stirred for 2 h at 20 °C using a shaker at 200 rpm (Green SSeriker, VS-203D; Vision Scientific Co., Ltd., Daejeon, Korea). After shaking for 2 h, the crude extract was filtered through a No.42 Whatman filter (110 mm paper diameter, GE Healthcare Life Science, Seoul, Korea) and the filtrate was evaporated using a rotary vacuum evaporator under 33 °C (EYELA N-1200A/CCA-1111; Tokyo Rikakikai Co., Ltd., Tokyo, Japan). The resulting residue of dried samples were reconstituted with 5 mL of 80% MeOH and filtered through a 0.22 µm PTFE membrane syringe filter (13 mm diameter, Thermo Scientific, Rochester, NY, USA). The extraction of PC was conducted in triplicate.

### 3.4. LC-MS/MS Analysis

The analysis of PC in RWSF and RSF samples were conducted using HPLC (high performance liquid chromatography) system equipped with electrospray ionization (ESI)—tandem mass spectrometer. The HPLC system was from Agilent Technologies (Santa Clara, CA, USA), which consisted of a 1200 Series Degasser (G1322A), a 1260 Infinity Quaternary Pump VL (G1311C), a 1100 Series Autosampler (G1313A), and a 1100 Series Column Compartment (G1316A). The HPLC column used for the separation of PC was from Thermo Syncronis C18 (4.6 × 150 mm, 5 µm). The mobile phase (MP) consisted of solvent A (0.1% formic acid in deionized water) and solvent B (0.1% formic acid in acetonitrile). The gradient conditions for mobile phases were as follows: control, 90% A/10% B; 10 min, 60% A/40% B; 20 min, 50% A/50% B; 25 min, 100% B; 26–30 min, 90% A/10% B for re-equilibrium. The flow rate was 0.5 mL/min and the column was maintained at room temperature. The injection volume was 10 µL and the analysis of each sample extract was conducted in triplicate.

An API 2000 system (AB Sciex, Framingham, MA, USA) equipped with an ESI (electrospray ionization) source and a MS/MS (triple-quadrupole mass spectrometer) was used to obtain the mass spectra. The analysis of ESI source was on negative ion and the MS/MS was in MRM modes. The optimum ESI source parameters were acquired by flow injection analysis (FIA) and they were as follows: curtain gas 50 psi, collision gas 2 psi, ion spray voltage—4400V, ion spray probe temperature 500 °C, nebulizer gas 40 psi, heating gas 50 psi, with the interface heater turned on. Nitrogen gas was used as curtain, collision, nebulizer, and heating gas. The position of the ion spray probe was aligned with 3 mm in vertical and 5 mm in horizontal axis. The optimum MRM parameters of each targeted PC and amino acid were determined by the infusion of corresponding authentic standard solution as given in the Supplementary Material as Table S1.

The PC and amino acids of samples were identified by comparing t_R_ and mass to charge ratio (*m/z*) of precursor and product ions (Q1 and Q3 values, respectively) with corresponding authentic standard solutions. The authentic standards of PC and amino acids were dissolved in the appropriate solvents according to their solubility to make stock solutions. The standard solutions were combined and then diluted in a series with 80% methanol for the calibration curve. The MRM ion chromatogram of the 56 selected PC and free amino acids along with mass spectra of the representative PC with their fragmentation patterns are shown in Supplementary Material as Figures S1–S3. The LOD and the limit of quantitation (LOQ) were calculated using the signal-to-noise (S/N) ratio at the level of 3 and 6, respectively (Supplementary Material as Table S2).

### 3.5. Statistical Analysis (SA)

The SA was performed using the SAS software (version 9.4; SAS Institute Inc., Cary, NC, USA). The data were analyzed using least significant difference (LSD) test and general linear model (GLM) at the 0.05 probability level.

## 4. Conclusions

This study explains the variation in the proportion of phenolic acids in RWSF and RSF over a year of storage. The total malonyl glucosides, total daidzeins, total isoflavones, acetyl glucosides, and aglycones are a few of the phenolic acids that showed changes, i.e., either decreased or increased in concentration. These changes were seen in RWSF and RSF, during control storage or longer storage and also based on the temperature of roasting and storage. Though many studies have already shown that processing and storage of food products can affect the constituents and nutritional value. This study aid in the elucidation of the proper methods for processing and storing of soybeans to retain their natural chemicals for the benefit of society.

## Figures and Tables

**Table 1:** Change of composition and content of phenolic compounds (PC) in raw soybean flour (RWSF) stored at 4, 20, and 45 °C (µg/g on dry weight basis).

Weeks		(1)	(2)	(3)	(4)	(5)	(6)	(7)	(8)	(9)	(10)	(11)	(12)	(13)	(14)	(15)	(16)	(17)	(18)	(19)	(20)
Control	RFT	0.14 ± 0.02 ^d^	0.48 ± 0.26 ^ef^	ND	0.04 ± 0.00 ^cd^	0.19 ± 0.03 ^b^	0.18 ± 0.03 ^c^	ND	ND	1.94 ± 0.10 ^d^	2.39 ± 0.44 ^d^	ND	15.39 ± 2.42 ^f^	2.38 ± 0.84 ^e^	<LOD	0.85 ± 0.29 ^f^	3.13 ± 0.43 ^g^	0.39 ± 0.04 ^c^	166.17 ± 4.47 ^d^	100.83 ± 4.28 ^e^	7.08 ± 0.68 ^d^
	RT	0.14 ± 0.02 ^d^	0.48 ± 0.26 ^ef^	ND	0.04 ± 0.00 ^cd^	0.19 ± 0.03 ^b^	0.18 ± 0.03 ^c^	ND	ND	1.94 ± 0.10 ^d^	2.39 ± 0.44 ^d^	ND	15.39 ± 2.42 ^f^	2.38 ± 0.84 ^e^	<LOD	0.85 ± 0.29 ^f^	3.13 ± 0.43 ^g^	0.39 ± 0.04 ^c^	166.17 ± 4.47 ^d^	100.83 ± 4.28 ^e^	7.08 ± 0.68 ^d^
	HT	0.14 ± 0.02 ^d^	0.48 ± 0.26 ^ef^	ND	0.04 ± 0.00 ^cd^	0.19 ± 0.03 ^b^	0.18 ± 0.03 ^c^	ND	ND	1.94 ± 0.10 ^d^	2.39 ± 0.44 ^d^	ND	15.39 ± 2.42 ^f^	2.38 ± 0.84 ^e^	<LOD	0.85 ± 0.29 ^f^	3.13 ± 0.43 ^g^	0.39 ± 0.04 ^c^	166.17 ± 4.47 ^d^	100.83 ± 4.28 ^e^	7.08 ± 0.68 ^d^
1	RFT	0.19 ± 0.01 ^b^	0.76 ± 0.16 ^cd^	ND	0.03 ± 0.00 ^e^	0.21 ± 0.02 ^a^	0.22 ± 0.02 ^b^	ND	ND	2.08 ± 0.05 ^bc^	3.42 ± 0.17 ^ab^	ND	18.01 ± 3.22 ^ef^	11.01 ± 2.46 ^d^	<LOD	3.03 ± 0.38 ^e^	6.20 ± 0.20 ^e^	0.53 ± 0.05 ^b^	212.39 ± 8.26 ^c^	127.67 ± 4.67 ^d^	9.59 ± 0.57 ^c^
	RT	0.18 ± 0.02 ^b^	0.85 ± 0.13 ^c^	ND	0.03 ± 0.00 ^c^	0.22 ± 0.01 ^a^	0.23 ± 0.02 ^b^	ND	ND	2.12 ± 0.08 ^b^	3.57 ± 0.14 ^ab^	ND	11.60 ± 2.55 ^f^	16.62 ± 1.83 ^e^	1.84 ± 0.25 ^c^	3.50 ± 0.29 ^e^	6.64 ± 0.46 ^f^	0.53 ± 0.06 ^e^	216.94 ± 13.06 ^c^	123.61 ± 7.07 ^d^	9.98 ± 0.55 ^d^
	HT	0.19 ± 0.01 ^bc^	0.79 ± 0.21 ^d^	ND	0.05 ± 0.00 ^b^	0.22 ± 0.02 ^a^	0.23 ± 0.02 ^cd^	ND	ND	2.17 ± 0.04 ^b^	3.15 ± 0.12 ^de^	ND	23.72 ± 6.30 ^f^	22.07 ± 2.30 ^e^	2.22 ± 0.21 ^de^	7.57 ± 0.58 ^f^	12.13 ± 0.71 ^f^	0.81 ± 0.10 ^e^	227.11 ± 9.88 ^d^	128.61 ± 3.82 ^c^	9.94 ± 0.54 ^d^
2	RFT	0.16 ± 0.01 ^c^	0.65 ± 0.15 ^de^	ND	0.05 ± 0.00 ^a^	0.21 ± 0.01 ^ab^	0.19 ± 0.02 ^c^	ND	ND	2.04 ± 0.08 ^c^	2.39 ± 0.47 ^d^	ND	21.86 ± 7.76 ^e^	11.03 ± 2.72 ^d^	2.24 ± 0.22 ^c^	3.89 ± 0.74 ^d^	7.23 ± 0.61 ^d^	0.64 ± 0.07 ^a^	220.33 ± 11.71 ^c^	138.89 ± 3.74 ^c^	9.84 ± 0.55 ^c^
	RT	0.15 ± 0.02 ^c^	0.59 ± 0.17 ^d^	ND	0.03 ± 0.00 ^c^	0.20 ± 0.02 ^b^	0.18 ± 0.02 ^c^	ND	ND	2.02 ± 0.07 ^bc^	3.39 ± 0.13 ^b^	ND	36.37 ± 5.56 ^e^	15.73 ± 2.42 ^e^	2.06 ± 0.26 ^c^	4.05 ± 0.42 ^d^	7.79 ± 0.40 ^e^	0.58 ± 0.06 ^de^	217.56 ± 10.42 ^c^	134.00 ± 2.37 ^c^	9.87 ± 0.54 ^d^
	HT	0.17 ± 0.02 ^cd^	0.64 ± 0.14 ^de^	ND	0.03 ± 0.00 ^d^	0.20 ± 0.01 ^b^	0.19 ± 0.01 ^e^	ND	ND	1.99 ± 0.08 ^de^	3.44 ± 0.12 ^bc^	ND	47.44 ± 7.46 ^e^	23.19 ± 2.22 ^e^	2.18 ± 0.20 ^e^	10.29 ± 0.57 ^e^	16.19 ± 0.50 ^e^	0.86 ± 0.03 ^e^	226.33 ± 10.07 ^d^	132.06 ± 3.81 ^c^	10.41 ± 0.61 ^cd^
4	RFT	0.18 ± 0.02 ^b^	0.89 ± 0.21 ^c^	ND	0.04 ± 0.01 ^bc^	0.22 ± 0.02 ^a^	0.24 ± 0.02 ^a^	ND	ND	2.13 ± 0.03 ^ab^	3.18 ± 0.40 ^b^	ND	58.44 ± 3.74 ^c^	24.59 ± 2.06 ^c^	2.43 ± 0.26 ^bc^	4.83 ± 0.99 ^c^	8.19 ± 0.96 ^c^	0.64 ± 0.08 ^a^	319.11 ± 33.13 ^b^	172.22 ± 10.09 ^b^	13.12 ± 0.62 ^b^
	RT	0.18 ± 0.02 ^b^	1.05 ± 0.24 ^b^	ND	0.03 ± 0.00 ^d^	0.18 ± 0.01 ^c^	0.24 ± 0.02 ^b^	ND	ND	2.05 ± 0.09 ^bc^	3.53 ± 0.10 ^ab^	ND	61.44 ± 4.65 ^c^	28.72 ± 1.93 ^c^	2.43 ± 0.30 ^b^	5.91 ± 0.42 ^c^	10.10 ± 0.38 ^c^	0.63 ± 0.07 ^cd^	294.78 ± 15.31 ^b^	164.89 ± 8.01 ^b^	12.70 ± 0.44 ^c^
	HT	0.18 ± 0.02 ^cd^	1.11 ± 0.15 ^c^	ND	0.03 ± 0.00 ^d^	0.13 ± 0.02 ^c^	0.24 ± 0.02 ^c^	ND	ND	2.07 ± 0.10 ^cd^	3.63 ± 0.13 ^b^	ND	79.56 ± 10.44 ^c^	38.15 ± 3.87 ^c^	2.41 ± 0.27 ^cd^	16.84 ± 0.52 ^d^	24.35 ± 0.83 ^d^	1.15 ± 0.08 ^d^	290.83 ± 20.87 ^a^	161.89 ± 7.59 ^b^	13.49 ± 0.68 ^b^
12	RFT	0.13 ± 0.01 ^d^	0.42 ± 0.10 ^f^	ND	0.04 ± 0.00 ^b^	0.17 ± 0.02 ^c^	0.18 ± 0.02 ^c^	ND	ND	1.79 ± 0.05 ^e^	2.73 ± 0.15 ^c^	ND	35.64 ± 4.20 ^d^	13.13 ± 2.05 ^d^	1.95 ± 0.30 ^d^	2.52 ± 0.90 ^e^	5.39 ± 1.07 ^f^	0.48 ± 0.07 ^b^	218.83 ± 9.21 ^c^	125.28 ± 3.17 ^d^	9.55 ± 0.40 ^c^
	RT	0.15 ± 0.01 ^c^	0.62 ± 0.17 ^d^	ND	0.04 ± 0.00 ^b^	0.11 ± 0.01 ^d^	0.18 ± 0.02 ^c^	ND	ND	1.81 ± 0.18 ^d^	2.84 ± 0.28 ^c^	ND	48.79 ± 7.24 ^d^	21.09 ± 2.77 ^d^	2.08 ± 0.28 ^c^	5.54 ± 0.57 ^c^	9.24 ± 0.23 ^d^	0.66 ± 0.05 ^c^	219.11 ± 13.55 ^c^	121.33 ± 5.16 ^d^	10.07 ± 0.64 ^d^
	HT	0.17 ± 0.02 ^d^	0.80 ± 0.08 ^d^	ND	0.05 ± 0.00 ^b^	0.09 ± 0.01 ^e^	0.22 ± 0.01 ^d^	ND	<LOD	2.00 ± 0.09 ^de^	3.08 ± 0.11 ^e^	ND	70.72 ± 4.76 ^d^	33.03 ± 2.08 ^d^	2.43 ± 0.15 ^c^	21.75 ± 1.01 ^c^	30.78 ± 0.50 ^c^	1.62 ± 0.14 ^c^	222.22 ± 7.22 ^d^	121.83 ± 3.20 ^d^	10.54 ± 0.42 ^c^
24	RFT	0.18 ± 0.01 ^b^	1.14 ± 0.21 ^b^	ND	0.03 ± 0.00 ^de^	0.12 ± 0.01 ^d^	0.24 ± 0.03 ^a^	ND	ND	1.94 ± 0.03 ^d^	3.17 ± 0.18 ^b^	ND	67.94 ± 10.50 ^b^	28.65 ± 5.47 ^b^	2.68 ± 0.31 ^b^	5.65 ± 0.50 ^b^	9.50 ± 0.74 ^b^	0.64 ± 0.02 ^a^	345.83 ± 24.99 ^a^	197.94 ± 16.25 ^a^	13.82 ± 0.91 ^a^
	RT	0.19 ± 0.01 ^b^	1.39 ± 0.12 ^a^	ND	0.03 ± 0.00 ^c^	0.10 ± 0.01 ^d^	0.26 ± 0.02 ^a^	ND	ND	2.09 ± 0.04 ^b^	3.38 ± 0.07 ^b^	ND	94.67 ± 5.76 ^b^	45.02 ± 2.16 ^b^	2.92 ± 0.16 ^a^	9.77 ± 0.39 ^b^	15.63 ± 0.32 ^b^	0.86 ± 0.05 ^b^	326.94 ± 23.54 ^a^	205.06 ± 11.44 ^a^	14.42 ± 0.79 ^a^
	HT	0.20 ± 0.01 ^b^	1.72 ± 0.14 ^b^	ND	0.04 ± 0.00 ^c^	0.08 ± 0.01 ^e^	0.29 ± 0.01 ^b^	ND	<LOD	2.13 ± 0.06 ^bc^	3.34 ± 0.19 ^cd^	ND	110.94 ± 5.88 ^b^	59.22 ± 3.07 ^b^	2.76 ± 0.15 ^b^	34.88 ± 1.12 ^b^	50.77 ± 1.07 ^b^	2.00 ± 0.09 ^b^	268.78 ± 16.17 ^b^	170.50 ± 5.35 ^a^	14.09 ± 0.51 ^a^
48	RFT	0.21 ± 0.02 ^a^	1.34 ± 0.30 ^a^	ND	0.03 ± 0.00 ^de^	0.06 ± 0.01 ^e^	0.25 ± 0.01 ^a^	ND	ND	2.15 ± 0.06 ^a^	3.70 ± 0.12 ^a^	ND	91.00 ± 7.77 ^a^	43.87 ± 2.96 ^a^	3.07 ± 0.19 ^a^	7.20 ± 0.90 ^a^	10.89 ± 0.97 ^a^	0.65 ± 0.04 ^a^	344.89 ± 29.87 ^a^	188.33 ± 20.91 ^a^	13.88 ± 0.78 ^a^
	RT	0.23 ± 0.02 ^a^	1.43 ± 0.23 ^a^	ND	0.05 ± 0.00 ^a^	0.08 ± 0.02 ^e^	0.28 ± 0.03 ^a^	ND	ND	2.24 ± 0.22 ^a^	3.75 ± 0.32 ^a^	ND	109.72 ± 8.43 ^a^	53.76 ± 4.33 ^a^	3.13 ± 0.40 ^a^	11.64 ± 0.71 ^a^	17.03 ± 0.69 ^a^	0.95 ± 0.05 ^a^	306.28 ± 17.62 ^b^	163.78 ± 4.79 ^b^	13.42 ± 0.81 ^b^
	HT	0.23 ± 0.02 ^a^	2.48 ± 0.26 ^a^	ND	0.06 ± 0.00 ^a^	0.10 ± 0.01 ^d^	0.38 ± 0.02 ^a^	ND	0.21 ± 0.03	2.44 ± 0.14 ^a^	4.18 ± 0.13 ^a^	1.52 ± 0.04	125.94 ± 9.65 ^a^	67.44 ± 2.60 ^a^	3.33 ± 0.27 ^a^	44.23 ± 1.26 ^a^	60.33 ± 1.58 ^a^	2.74 ± 0.18 ^a^	255.00 ± 9.68 ^c^	158.50 ± 4.42 ^b^	13.14 ± 0.35 ^b^

(1): Protocatechuic acid; (2): *p*-Hydroxybenzoic acid; (3): Syringic acid; (4): Salicylic acid; (5): *β*-Resorcylic acid; (6): *p*-Coumaric acid; (7): Chlorogenic acid; (8): Ferulic acid; (9): Daidzein; (10): Genistein; (11): Glycitein; (12): Daidzin; (13): Genistin; (14): Glycitin; (15): Acetyldaidzin; (16): Acetylgenistin; (17): Acetylglycitin; (18): Malonyldaidzin; (19): Malonylgenistin; (20): Malonylglycitin. RFT: Refrigerated temperature (4 °C); RT: Room temperature (20 °C); HT: High temperature (45 °C). ND: not detected, <LOD: below the limit of detection. The values presented are the mean ± SEM. ^a–f^ The superscript in each mean represents significant *p* values (*p* < 0.05) that are different from each other.

**Table 2:** Change of composition and content of PC in roasted soybean flour stored at 4, 20, and 45 °C (µg/g on dry weight basis).

Weeks		(1)	(2)	(3)	(4)	(5)	(6)	(7)	(8)	(9)	(10)	(11)	(12)	(13)	(14)	(15)	(16)	(17)	(18)	(19)	(20)
Control	RFT	0.20 ± 0.01 ^cd^	0.72 ± 0.14 ^d^	22.40 ± 1.06 ^e^	0.13 ± 0.01 ^b^	0.12 ± 0.04 ^d^	0.96 ± 0.09 ^ab^	0.13 ± 0.01 ^b^	1.24 ± 0.14 ^c^	2.99 ± 0.09 ^d^	4.97 ± 0.32 ^f^	3.13 ± 0.14 ^e^	57.47 ± 5.60 ^f^	41.83 ± 4.68 ^d^	<LOD	54.33 ± 3.45 ^e^	61.00 ± 2.84 ^e^	5.86 ± 0.24 ^e^	<LOD	<LOD	ND
	RT	0.20 ± 0.01 ^cd^	0.72 ± 0.14 ^d^	22.40 ± 1.06 ^e^	0.13 ± 0.01 ^b^	0.12 ± 0.04 ^d^	0.96 ± 0.09 ^ab^	0.13 ± 0.01 ^b^	1.24 ± 0.14 ^c^	2.99 ± 0.09 ^d^	4.97 ± 0.32 ^f^	3.13 ± 0.14 ^e^	57.47 ± 5.60 ^f^	41.83 ± 4.68 ^d^	<LOD	54.33 ± 3.45 ^e^	61.00 ± 2.84 ^e^	5.86 ± 0.24 ^e^	<LOD	<LOD	ND
	HT	0.20 ± 0.01 ^cd^	0.72 ± 0.14 ^d^	22.40 ± 1.06 ^e^	0.13 ± 0.01 ^b^	0.12 ± 0.04 ^d^	0.96 ± 0.09 ^ab^	0.13 ± 0.01 ^b^	1.24 ± 0.14 ^c^	2.99 ± 0.09 ^d^	4.97 ± 0.32 ^f^	3.13 ± 0.14 ^e^	57.47 ± 5.60 ^f^	41.83 ± 4.68 ^d^	<LOD	54.33 ± 3.45 ^e^	61.00 ± 2.84 ^e^	5.86 ± 0.24 ^e^	<LOD	<LOD	ND
1	RFT	0.22 ± 0.02 ^b^	1.08 ± 0.15 ^c^	28.14 ± 3.05 ^c^	0.14 ± 0.01 ^a^	0.17 ± 0.01 ^b^	1.00 ± 0.07 ^a^	0.14 ± 0.02 ^ab^	1.31 ± 0.08 ^c^	3.18 ± 0.08 ^c^	5.50 ± 0.27 ^e^	3.32 ± 0.11 ^d^	70.28 ± 7.56 ^e^	59.94 ± 3.89 ^c^	2.49 ± 0.21 ^b^	66.28 ± 2.94 ^d^	77.50 ± 3.80 ^d^	6.94 ± 0.26 ^b^	<LOD	<LOD	ND
	RT	0.23 ± 0.02 ^cd^	1.51 ± 0.14 ^c^	29.18 ± 2.49 ^bc^	0.13 ± 0.01 ^b^	0.17 ± 0.01 ^bc^	0.96 ± 0.06 ^bcd^	0.12 ± 0.02 ^cd^	1.34 ± 0.13 ^bcd^	3.14 ± 0.08 ^c^	6.93 ± 0.20 ^d^	3.39 ± 0.14 ^c^	77.94 ± 4.55 ^e^	63.22 ± 4.51 ^e^	2.67 ± 0.17 ^b^	70.44 ± 2.89 ^d^	81.17 ± 2.51 ^d^	6.72 ± 0.24 ^b^	<LOD	<LOD	ND
	HT	0.24 ± 0.02 ^c^	1.85 ± 0.22 ^d^	29.84 ± 3.74 ^a^	0.13 ± 0.01 ^d^	0.17 ± 0.01 ^c^	0.97 ± 0.08 ^b^	0.13 ± 0.02 ^bc^	1.39 ± 0.09 ^b^	3.15 ± 0.10 ^de^	7.14 ± 0.29 ^c^	3.57 ± 0.10 ^ab^	88.00 ± 7.61 ^e^	67.72 ± 4.06 ^b^	2.80 ± 0.30 ^ab^	71.28 ± 1.79 ^b^	80.22 ± 1.50 ^c^	6.69 ± 0.33 ^b^	<LOD	<LOD	ND
2	RFT	0.22 ± 0.02 ^b^	1.04 ± 0.27 ^c^	25.06 ± 1.88 ^de^	0.13 ± 0.01 ^b^	0.18 ± 0.01 ^b^	0.89 ± 0.12 ^b^	0.13 ± 0.02 ^b^	1.25 ± 0.12 ^c^	3.15 ± 0.09 ^c^	6.76 ± 0.54 ^d^	3.34 ± 0.19 ^d^	87.67 ± 9.08 ^d^	63.44 ± 5.12 ^c^	2.44 ± 0.31 ^b^	67.28 ± 2.55 ^d^	77.78 ± 3.16 ^d^	6.83 ± 0.28 ^bc^	<LOD	<LOD	ND
	RT	0.22 ± 0.02 ^de^	1.41 ± 0.22 ^c^	28.13 ± 2.65 ^c^	0.11 ± 0.01 ^e^	0.19 ± 0.01 ^a^	0.90 ± 0.09 ^d^	0.12 ± 0.03 ^cd^	1.45 ± 0.08 ^ab^	3.22 ± 0.10 ^c^	7.68 ± 0.40 ^bc^	3.53 ± 0.21 ^c^	106.56 ± 12.97 ^cd^	72.33 ± 4.71 ^d^	2.55 ± 0.21 ^b^	72.78 ± 3.71 ^d^	83.17 ± 2.54 ^d^	6.83 ± 0.36 ^b^	<LOD	<LOD	ND
	HT	0.25 ± 0.01 ^c^	1.95 ± 0.28 ^d^	30.14 ± 2.38 ^a^	0.12 ± 0.01 ^e^	0.18 ± 0.02 ^bc^	0.95 ± 0.05 ^b^	0.13 ± 0.03 ^bc^	1.42 ± 0.13 ^ab^	3.23 ± 0.09 ^cd^	7.68 ± 0.18 ^b^	3.66 ± 0.15 ^a^	119.00 ± 8.56 ^b^	75.78 ± 2.06 ^a^	2.88 ± 0.25 ^ab^	78.44 ± 8.03 ^a^	87.22 ± 3.08 ^a^	7.34 ± 1.10 ^a^	<LOD	<LOD	ND
4	RFT	0.20 ± 0.02 ^d^	1.14 ± 0.25 ^c^	36.26 ± 4.14 ^a^	0.11 ± 0.01 ^c^	0.11 ± 0.01 ^d^	0.96 ± 0.08 ^ab^	0.14 ± 0.02 ^b^	1.68 ± 0.16 ^a^	3.30 ± 0.09 ^b^	7.74 ± 0.25 ^b^	3.82 ± 0.19 ^b^	119.39 ± 9.14 ^b^	80.50 ± 5.78 ^b^	2.63 ± 0.37 ^b^	77.28 ± 3.02 ^c^	89.28 ± 2.71 ^c^	6.20 ± 0.20 ^d^	<LOD	<LOD	ND
	RT	0.21 ± 0.01 ^ef^	1.54 ± 0.22 ^c^	31.67 ± 3.87 ^ab^	0.10 ± 0.00 ^f^	0.12 ± 0.01 ^d^	0.98 ± 0.05 ^bc^	0.11 ± 0.02 ^d^	1.47 ± 0.16 ^a^	3.46 ± 0.14 ^b^	7.93 ± 0.27 ^b^	3.76 ± 0.15 ^b^	116.22 ± 9.97 ^c^	78.72 ± 3.68 ^c^	2.47 ± 0.25 ^b^	77.00 ± 2.06 ^c^	89.72 ± 3.95 ^c^	6.06 ± 0.33 ^d^	<LOD	<LOD	ND
	HT	0.22 ± 0.02 ^d^	2.05 ± 0.29 ^d^	30.29 ± 3.94 ^a^	0.10 ± 0.00 ^f^	0.13 ± 0.02 ^d^	1.10 ± 0.07 ^a^	0.15 ± 0.01 ^ab^	1.51 ± 0.10 ^a^	3.42 ± 0.13 ^b^	7.96 ± 0.16 ^a^	3.61 ± 0.15 ^a^	114.28 ± 8.87 ^bc^	76.89 ± 4.98 ^a^	2.62 ± 0.39 ^bc^	77.56 ± 3.02 ^a^	87.89 ± 3.05 ^a^	5.74 ± 0.27 ^c^	<LOD	<LOD	ND
12	RFT	0.19 ± 0.01 ^d^	1.02 ± 0.24 ^c^	26.72 ± 2.30 ^cd^	0.14 ± 0.01 ^a^	0.15 ± 0.02 ^c^	0.95 ± 0.09 ^ab^	0.13 ± 0.02 ^b^	1.25 ± 0.09 ^c^	2.93 ± 0.07 ^d^	5.04 ± 0.49 ^f^	3.32 ± 0.08 ^d^	98.33 ± 6.84 ^c^	62.67 ± 1.41 ^c^	2.62 ± 0.28 ^b^	65.44 ± 1.99 ^d^	77.33 ± 0.66 ^d^	6.62 ± 0.33 ^c^	<LOD	<LOD	ND
	RT	0.24 ± 0.01 ^c^	1.88 ± 0.36 ^b^	27.13 ± 3.02 ^c^	0.14 ± 0.00 ^a^	0.17 ± 0.01 ^c^	0.91 ± 0.08 ^cd^	0.16 ± 0.02 ^ab^	1.25 ± 0.12 ^cd^	3.01 ± 0.09 ^d^	5.35 ± 0.41 ^e^	3.40 ± 0.16 ^c^	105.00 ± 7.25 ^d^	65.22 ± 5.78 ^e^	2.71 ± 0.44 ^b^	66.72 ± 2.18 ^e^	81.61 ± 5.25 ^d^	6.71 ± 0.31 ^b^	<LOD	<LOD	ND
	HT	0.25 ± 0.02 ^c^	2.88 ± 0.33 ^c^	27.96 ± 1.56 ^ab^	0.15 ± 0.00 ^c^	0.16 ± 0.01 ^c^	0.93 ± 0.06 ^b^	0.10 ± 0.01 ^d^	1.33 ± 0.10 ^bc^	3.13 ± 0.04 ^e^	6.32 ± 0.31 ^d^	3.47 ± 0.10 ^b^	106.39 ± 6.43 ^d^	69.94 ± 3.26 ^b^	2.76 ± 0.32 ^abc^	69.44 ± 2.16 ^b^	83.94 ± 3.10 ^b^	6.59 ± 0.13 ^b^	<LOD	<LOD	ND
24	RFT	0.22 ± 0.01 ^bc^	1.78 ± 0.18 ^b^	32.14 ± 3.60 ^b^	0.11 ± 0.01 ^c^	0.17 ± 0.01 ^b^	1.01 ± 0.05 ^a^	0.15 ± 0.02 ^a^	1.43 ± 0.09 ^b^	3.32 ± 0.06 ^b^	7.31 ± 0.13 ^c^	3.68 ± 0.11 ^c^	157.83 ± 12.36 ^a^	101.06 ± 6.23 ^a^	3.34 ± 0.30 ^a^	90.78 ± 3.52 ^b^	111.39 ± 4.62 ^a^	7.42 ± 0.48 ^a^	<LOD	<LOD	ND
	RT	0.28 ± 0.02 ^b^	2.97 ± 0.27 ^a^	33.14 ± 2.48 ^a^	0.12 ± 0.01 ^cd^	0.19 ± 0.01 ^ab^	1.01 ± 0.04 ^ab^	0.17 ± 0.02 ^a^	1.37 ± 0.11 ^abc^	3.38 ± 0.11 ^b^	7.49 ± 0.25 ^c^	3.80 ± 0.15 ^b^	170.72 ± 14.98 ^a^	103.39 ± 4.49 ^b^	3.06 ± 0.19 ^a^	95.89 ± 1.95 ^a^	111.28 ± 2.43 ^a^	7.67 ± 0.26 ^a^	<LOD	<LOD	ND
	HT	0.47 ± 0.02 ^b^	14.11 ± 1.01 ^b^	26.66 ± 1.20 ^b^	0.21 ± 0.01 ^b^	0.20 ± 0.02 ^b^	0.95 ± 0.05 ^b^	0.16 ± 0.01 ^a^	1.02 ± 0.09 ^d^	3.27 ± 0.07 ^c^	7.14 ± 0.17 ^c^	3.00 ± 0.11 ^d^	128.17 ± 6.12 ^a^	61.22 ± 3.29 ^c^	2.92 ± 0.21 ^a^	79.22 ± 1.94 ^a^	80.72 ± 1.68 ^c^	6.59 ± 0.17 ^b^	ND	<LOD	ND
48	RFT	0.25 ± 0.02 ^a^	2.03 ± 0.34 ^a^	35.51 ± 2.96 ^a^	0.13 ± 0.01 ^b^	0.22 ± 0.01 ^a^	1.02 ± 0.06 ^a^	0.14 ± 0.02 ^ab^	1.52 ± 0.08 ^b^	3.69 ± 0.12 ^a^	8.62 ± 0.22 ^a^	4.27 ± 0.12 ^a^	156.56 ± 12.57 ^a^	102.33 ± 4.56 ^a^	3.20 ± 0.16 ^a^	94.06 ± 3.74 ^a^	107.39 ± 4.20 ^b^	7.05 ± 0.24 ^b^	<LOD	<LOD	ND
	RT	0.30 ± 0.02 ^a^	2.99 ± 0.31 ^a^	33.44 ± 2.75 ^a^	0.12 ± 0.00 ^d^	0.20 ± 0.01 ^a^	1.07 ± 0.08 ^a^	0.15 ± 0.02 ^b^	1.48 ± 0.14 ^a^	3.85 ± 0.10 ^a^	8.58 ± 0.22 ^a^	4.03 ± 0.14 ^a^	159.22 ± 13.73 ^b^	108.06 ± 2.26 ^a^	3.18 ± 0.45 ^a^	91.00 ± 2.55 ^b^	103.78 ± 5.19 ^b^	6.41 ± 0.19 ^c^	<LOD	<LOD	ND
	HT	0.72 ± 0.03 ^a^	17.24 ± 1.55 ^a^	22.58 ± 2.69 ^c^	0.27 ± 0.01 ^a^	0.22 ± 0.02 ^a^	1.06 ± 0.08 ^a^	0.13 ± 0.03 ^bc^	0.88 ± 0.08 ^e^	3.67 ± 0.08 ^a^	7.87 ± 0.25 ^ab^	3.14 ± 0.10 ^c^	109.56 ± 12.09 ^cd^	40.06 ± 2.40 ^d^	2.49 ± 0.25 ^c^	69.72 ± 2.08 ^b^	79.00 ± 2.32 ^c^	5.61 ± 0.22 ^c^	ND	<LOD	ND

(1): Protocatechuic acid; (2): *p*-Hydroxybenzoic acid; (3): Syringic acid; (4): Salicylic acid; (5): *β*-Resorcylic acid; (6): *p*-Coumaric acid; (7): Chlorogenic acid; (8): Ferulic acid; (9): Daidzein; (10): Genistein; (11): Glycitein; (12): Daidzin; (13): Genistin; (14): Glycitin; (15): Acetyldaidzin; (16): Acetylgenistin; (17): Acetylglycitin; (18): Malonyldaidzin; (19): Malonylgenistin; (20): Malonylglycitin. (RFT): Refrigerated temperature (4 °C); (RT): Room temperature (20 °C); (HT): High temperature (45 °C). ND: not detected, <LOD: below the limit of detection. The values presented are the mean ± SEM. ^a–f^ The superscript in each mean represents significant *p* values (*p* < 0.05) that are different from each other.

**Table 3:** Total phenolic acids and isoflavones in RWSF and RSF at different storage time and temperature.

	Raw Soybean Flour			Roasted Soybean Flour		
Storage Time (Weeks)	Total Phenolic Acids	Total Isoflavones	Sum of Phenolic Acids and Isoflavones	Total Phenolic Acids	Total Isoflavones	Sum of Phenolic Acids and Isoflavones
Refrigerated Temperature (4 °C)						
Control	1.04 ± 0.31 ^e^	300.55 ± 9.22 ^e^	301.59 ± 9.48 ^e^	25.88 ± 1.29 ^e^	231.58 ± 13.74 ^e^	257.47 ± 14.42 ^e^
1	1.41 ± 0.18 ^cd^	393.92 ± 13.14 ^d^	395.34 ± 13.14 ^d^	32.20 ± 3.02 ^c^	295.44 ± 9.98 ^d^	327.64 ± 10.37 ^d^
2	1.27 ± 0.16 ^d^	420.39 ± 10.12 ^d^	421.66 ± 10.16 ^d^	28.89 ± 2.03 ^d^	318.68 ± 16.14 ^c^	347.58 ± 17.19 ^c^
4	1.57 ± 0.21 ^bc^	608.90 ± 35.00 ^c^	610.48 ± 35.15 ^c^	40.58 ± 4.27 ^a^	390.13 ± 6.98 ^b^	430.71 ± 8.31 ^b^
12	0.95 ± 0.10 ^e^	417.30 ± 12.15 ^d^	418.25 ± 12.09 ^d^	30.55 ± 2.31 ^cd^	324.30 ± 7.50 ^c^	354.85 ± 8.93 ^c^
24	1.72 ± 0.23 ^ab^	677.77 ± 46.58 ^b^	679.49 ± 46.76 ^b^	37.00 ± 3.76 ^b^	486.13 ± 12.54 ^a^	523.13 ± 13.99 ^a^
48	1.90 ± 0.32 ^a^	709.65 ± 45.48 ^a^	711.55 ± 45.72 ^a^	40.82 ± 3.02 ^a^	487.16 ± 18.98 ^a^	527.98 ± 20.63 ^a^
Room Temperature (20 °C)						
Control	1.04 ± 0.31 ^c^	300.55 ± 9.22 ^f^	301.59 ± 9.48 ^f^	25.88 ± 1.29 ^e^	231.58 ± 13.74 ^e^	257.47 ± 14.42 ^e^
1	1.51 ± 0.13 ^b^	396.96 ± 16.52 ^e^	398.47 ± 16.52 ^e^	33.64 ± 2.57 ^bc^	315.63 ± 9.71 ^f^	349.27 ± 10.38 ^f^
2	1.15 ± 0.19 ^c^	433.42 ± 13.45 ^d^	434.57 ± 13.49 ^d^	32.54 ± 2.67 ^c^	358.64 ± 17.14 ^d^	391.18 ± 18.90 ^d^
4	1.67 ± 0.24 ^b^	587.17 ± 20.61 ^c^	588.84 ± 20.62 ^c^	36.20 ± 4.01 ^b^	385.34 ± 13.93 ^c^	421.54 ± 14.10 ^c^
12	1.10 ± 0.19 ^c^	442.58 ± 27.69 ^d^	443.67 ± 27.80 ^d^	31.89 ± 3.34 ^c^	339.74 ± 16.75 ^e^	371.63 ± 18.45 ^e^
24	1.97 ± 0.14 ^a^	720.76 ± 26.01 ^a^	722.73 ± 25.92 ^a^	39.25 ± 2.48 ^a^	506.68 ± 18.17 ^a^	545.93 ± 18.03 ^a^
48	2.06 ± 0.25 ^a^	685.68 ± 20.68 ^b^	687.74 ± 20.55 ^b^	39.74 ± 2.77 ^a^	488.11 ± 17.60 ^b^	527.85 ± 18.47 ^b^
High Temperature (45 °C)						
Control	1.04 ± 0.31 ^f^	300.55 ± 9.22 ^g^	301.59 ± 9.48 ^g^	25.88 ± 1.29 ^e^	231.58 ± 13.74 ^e^	257.47 ± 14.42 ^e^
1	1.47 ± 0.21 ^d^	439.50 ± 18.56 ^f^	440.97 ± 18.64 ^f^	34.73 ± 3.84 ^b^	330.57 ± 8.83 ^d^	365.30 ± 10.97 ^c^
2	1.24 ± 0.15 ^e^	474.37 ± 16.15 ^e^	475.61 ± 16.21 ^e^	35.14 ± 2.34 ^b^	385.23 ± 15.40 ^a^	420.37 ± 15.33 ^a^
4	1.70 ± 0.17 ^c^	634.35 ± 40.58 ^c^	636.05 ± 40.48 ^c^	35.55 ± 4.00 ^b^	379.96 ± 14.28 ^ab^	415.51 ± 13.90 ^a^
12	1.33 ± 0.08 ^de^	520.02 ± 10.21 ^d^	521.35 ± 10.25 ^d^	33.76 ± 1.70 ^b^	352.01 ± 8.45 ^c^	385.77 ± 8.70 ^b^
24	2.32 ± 0.13 ^b^	719.41 ± 20.25 ^b^	721.74 ± 20.26 ^b^	43.76 ± 2.08 ^a^	372.26 ± 8.00 ^b^	416.02 ± 7.54 ^a^
48	3.45 ± 0.25 ^a^	738.80 ± 7.14 ^a^	742.25 ± 7.24 ^a^	43.09 ± 3.73 ^a^	321.12 ± 10.41 ^d^	364.21 ± 12.65 ^c^

The values presented are the mean ± SEM. The superscript in each mean represents significant *p* values (*p* < 0.05) that are different from each other.

**Table 4:** Comparison of PC composition and content (µg/g on dry weight basis) depending on soybean flour type, storage condition, and duration and the associated GLM *p*-value for main factor and its interactions.

PC	Soybean Flour Type (T)		Storage Time (°C)			Storage Time (D)							*p*-Value						
	RWSF	RSF	4	20	45	Control	1 Week	2 Weeks	4 Weeks	12 Weeks	24 Weeks	48 Weeks	Main Factor			Interaction			
	n = 63	n = 63	n = 42	n = 42	n = 42	n = 18	n = 18	n = 18	n = 18	n = 18	n = 18	n = 18	T	C	D	T*C	T*D	C*D	T*C*D
Protocatechuic acid	0.18 ^b^	0.27 ^a^	0.19 ^c^	0.21 ^b^	0.26 ^a^	0.17 ^f^	0.21 ^c^	0.20 ^d^	0.20 ^d^	0.19 ^e^	0.26 ^b^	0.32 ^a^	****	****	****	****	****	****	****
*p*-Hydroxybenzoic acid	1.01 ^b^	3.22 ^a^	1.04 ^c^	1.39 ^b^	3.49 ^a^	0.60 ^f^	1.14 d^e^	1.05 ^e^	1.30 ^c^	1.27 ^cd^	3.85 ^b^	4.58 ^a^	****	****	****	****	****	****	****
Syringic acid	ND	29.28	29.46 ^a^	29.30 ^a^	27.12 ^b^	22.40 ^f^	29.05 ^cd^	27.77 ^de^	32.74 ^a^	27.27 ^e^	30.65 ^b^	30.51 ^bc^	-	****	****	-	-	****	-
Salicylic acid	0.04 ^b^	0.14 ^a^	0.08 ^b^	0.08 ^c^	0.10 ^a^	0.08 ^e^	0.09 ^d^	0.08 ^e^	0.07 ^f^	0.09 ^b^	0.09 ^c^	0.11 ^a^	****	****	****	****	****	****	****
*β*-Resorcylic acid	0.15 ^b^	0.17 ^a^	0.17 ^a^	0.16 ^b^	0.16 ^b^	0.16 ^b^	0.20 ^a^	0.19 ^a^	0.15 ^c^	0.14 ^c^	0.14 ^c^	0.15 ^c^	****	**	****	****	****	****	****
*p*-Coumaric acid	0.23 ^b^	0.98 ^a^	0.59 ^b^	0.60 ^b^	0.62 ^a^	0.57 ^d^	0.60 ^c^	0.55 ^e^	0.63 ^b^	0.56 ^de^	0.63 ^b^	0.68 ^a^	****	***	****	ns	ns	**	****
Chlorogenic acid	ND	0.14	0.14 ^a^	0.14 ^a^	0.13 ^a^	0.13 ^c^	0.13 ^bc^	0.13 ^c^	0.13 ^c^	0.13 ^c^	0.16 ^a^	0.14 ^b^	-	ns	****	-	-	****	-
Ferulic acid	0.21 ^b^	1.35 ^a^	1.38 ^a^	1.37 ^a^	1.12 ^b^	1.24 ^c^	1.35 ^b^	1.37 ^b^	1.55 ^a^	1.28 ^c^	1.27 ^c^	1.02 ^d^	****	****	****	-	-	****	-
Daidzein	2.06 ^b^	3.29 ^a^	2.62 ^b^	2.67 ^a^	2.69 ^a^	2.46 ^e^	2.64 ^d^	2.61 ^d^	2.74 ^b^	2.45 ^e^	2.69 ^c^	3.01 ^a^	****	****	****	***	****	****	****
Genistein	3.28 ^b^	7.05 ^a^	4.78 ^b^	5.13 ^a^	5.16 ^a^	3.68 ^f^	4.95 ^d^	5.22 ^c^	5.66 ^b^	4.23 ^e^	5.31 ^c^	6.12 ^a^	****	****	****	ns	****	****	****
Glycitein	1.52 ^b^	3.54 ^a^	3.55 ^a^	3.58 ^a^	3.14 ^b^	3.13 ^f^	3.43 ^cd^	3.51 ^b^	3.73 ^a^	3.40 ^d^	3.50 ^bc^	3.24 ^e^	****	****	****	-	-	****	-
Daidzin	59.43 ^b^	113.08 ^a^	75.42 ^b^	83.65 ^a^	85.47 ^a^	36.43 ^g^	48.26 ^f^	69.81 ^e^	91.56 ^c^	77.48 ^d^	121.71 ^b^	125.33 ^a^	****	****	****	****	****	****	****
Genistin	29.41 ^b^	73.39 ^a^	46.17 ^c^	51.15 ^a^	48.50 ^b^	22.11 ^f^	40.10 ^e^	43.58 ^d^	54.60 ^c^	44.18 ^d^	66.43 ^b^	69.25 ^a^	****	****	****	****	****	****	****
Glycitin	2.48 ^b^	2.77 ^a^	2.64 ^a^	2.59 ^a^	2.65 ^a^	ND	2.40 ^cd^	2.39 ^d^	2.50 ^c^	2.43 ^cd^	2.95 ^b^	3.07 ^a^	****	ns	****	**	****	****	****
Acetyldaidzin	10.73 ^b^	75.52 ^a^	38.82 ^c^	40.67 ^b^	45.46 ^a^	27.59 ^e^	37.02 ^d^	39.46 ^c^	43.24 ^b^	38.57 ^c^	52.70 ^a^	52.98 ^a^	****	****	****	****	****	****	****
Acetylgenistin	16.40 ^b^	86.92 ^a^	46.59 ^c^	48.66 ^b^	54.12 ^a^	32.07 ^f^	43.98 ^e^	46.56 ^d^	51.59 ^b^	48.05 ^c^	63.21 ^a^	63.07 ^a^	****	****	****	****	****	****	****
Acetylglycitin	0.91 ^b^	6.63 ^a^	3.64 ^b^	3.63 ^b^	3.86 ^a^	3.12 ^f^	3.70 ^d^	3.85 ^bc^	3.40 ^e^	3.78 ^cd^	4.20 ^a^	3.90 ^b^	****	****	****	****	****	****	****
Malonyldaidzin	257.87	ND	261.08 ^a^	249.68 ^b^	236.63 ^c^	166.17 ^d^	218.81 ^c^	221.41 ^c^	301.57 ^b^	220.06 ^c^	313.85 ^a^	302.06 ^b^	-	****	****	-	-	****	-
Malonylgenistin	149.33	ND	150.17 ^a^	144.79 ^b^	139.17 ^c^	100.83 ^e^	126.63 ^d^	134.98 ^c^	166.33 ^b^	122.81 ^d^	191.17 ^a^	170.20 ^b^	-	****	****	-	-	****	-
Malonylglycitin	11.53	ND	10.99 ^b^	11.08 ^ab^	11.24 ^a^	7.08 ^e^	9.84 ^d^	10.04 ^d^	13.10 ^c^	10.05 ^d^	14.11 ^a^	13.48 ^b^	-	ns	****	-	-	**	-
Total phenolic acids	1.62 ^b^	35.54 ^a^	17.56 ^b^	17.83 ^b^	18.89 ^a^	13.46 ^f^	17.49 ^d^	16.70 ^e^	19.55 ^c^	16.60 ^e^	21.00 ^b^	21.84 ^a^	****	****	****	***	****	****	****
Total isoflavones	543.24 ^a^	372.04 ^b^	432.99 ^b^	442.35 ^a^	442.84 ^a^	266.07 ^f^	362.00 ^e^	398.46 ^d^	497.64 ^c^	399.32 ^d^	580.50 ^a^	571.75 ^b^	****	****	****	****	****	****	****
Sum of phenolic acids and isoflavones	544.86 ^a^	407.58 ^b^	450.55 ^b^	460.18 ^a^	461.73 ^a^	279.53 ^f^	379.50 ^e^	415.16 ^d^	517.19 ^c^	415.92 ^d^	601.51 ^a^	593.60 ^b^	****	****	****	****	****	****	****

^a–g^ Values with different superscripts differ significantly with respect to soybean flour type, storage time, or temperature (*p* < 0.05). ND: not detected, ns: non-significant, * *p* < 0.05, ** *p* < 0.01, *** *p* < 0.001, **** *p* < 0.0001.

## Supplementary Materials

Change of composition and content of phenolic compounds of RSF at room and high temperature, MRM ion chromatogram of standards, MS/MS spectra of phenolic acids and isoflavones are available online.
