# Supplementary material for: Effect of Storage Time and Temperature on Phenolic Compounds of Soybean (Glycine max L.) Flour
Source: Molecules. 2018 Sep 5;23(9):2269. doi: 10.3390/molecules23092269 (PMC6225263; doi:10.3390/molecules23092269)
Supplement: Supplementary file 1 [file molecules-23-02269-s001.pdf]

Electronic Supplementary Information (ESI) for molecules

Effect of Storage Time and Temperature on Phenolic Compounds of Soybean (*Glycine max* L.) Flour

Mayakrishnan Prabakaran [1], Ji-Hee Lee [1], Ateeque Ahmad [2], Seung-Hyun Kim [1], Koan-Sik Woo [3], Mi-Jung Kim [4] and Ill-Min Chung [1,*]

[1] Department of Crop Science, College of Sanghuh Life Science, Konkuk University, 120 Neungdong-ro, Gwangjin-gu, Seoul 05029, Republic of Korea; prabakarannitt@gmail.com (M.P.); j1225@konkuk.ac.kr (J.-H.L.); kshkim@konkuk.ac.kr (S.-H.K.)

[2] Process Chemistry and Technology Department, CSIR-Central Institute of Medicinal and Aromatic Plants, Lucknow 226015, India; ateeque97@gmail.com

[3] Department of Central Area Crop Science, National Institute of Crop Science, Rural Development Administration, Suwon 16429, Republic of Korea; wooks@korea.kr

[4] Department of Central Area Crop Science, National Institute of Crop Science, RDA, Suwon 16613, Republic of Korea; tyche@korea.kr

* Correspondence: imcim@konkuk.ac.kr; Tel.: +82-02-450-3730; Fax: +82-02-446-7856

Table S1. Change of composition and content of phenolic compounds in roasted soybean flour stored at room temperature (μg/g on dry weight basis).

	Storage duration						
	Initial	1 week	2 weeks	4 weeks	12 weeks	24 weeks	48 weeks
Protocatechuic acid	0.20 ± 0.01^{f}	0.23 ± 0.02^{cd}	0.22 ± 0.02^{de}	0.21 ± 0.01^{ef}	0.24 ± 0.01^{c}	0.28 ± 0.02^{b}	0.30 ± 0.02^{a}
p-Hydroxybenzoic acid	0.72 ± 0.14^{d}	1.51 ± 0.14^{c}	1.41 ± 0.22^{c}	1.54 ± 0.22^{c}	1.88 ± 0.36^{b}	2.97 ± 0.27^{a}	2.99 ± 0.31^{a}
Syringic acid	22.40 ± 1.06^{d}	29.18 ± 2.49^{bc}	28.13 ± 2.65^{c}	31.67 ± 3.87^{ab}	27.13 ± 3.02^{c}	33.14 ± 2.48^{a}	33.44 ± 2.75^{a}
Salicylic acid	0.13 ± 0.01^{bc}	0.13 ± 0.01^{b}	0.11 ± 0.01^{e}	0.10 ± 0.00^{f}	0.14 ± 0.00^{a}	0.12 ± 0.01^{cd}	0.12 ± 0.00^{d}
β-Resorcylic acid	0.12 ± 0.04^{d}	0.17 ± 0.01^{bc}	0.19 ± 0.01^{a}	0.12 ± 0.01^{d}	0.17 ± 0.01^{c}	0.19 ± 0.01^{ab}	0.20 ± 0.01^{a}
p-Coumaric acid	0.96 ± 0.09^{bcd}	0.96 ± 0.06^{bcd}	0.90 ± 0.09^{d}	0.98 ± 0.05^{bc}	0.91 ± 0.08^{cd}	1.01 ± 0.04^{ab}	1.07 ± 0.08^{a}
Chlorogenic acid	0.13 ± 0.01^{c}	0.12 ± 0.02^{cd}	0.12 ± 0.03^{cd}	0.11 ± 0.02^{d}	0.16 ± 0.02^{ab}	0.17 ± 0.02^{a}	0.15 ± 0.02^{b}
Ferulic acid	1.24 ± 0.14^{d}	1.34 ± 0.13^{bcd}	1.45 ± 0.08^{ab}	1.47 ± 0.16^{a}	1.25 ± 0.12^{cd}	1.37 ± 0.11^{abc}	1.48 ± 0.14^{a}
Daidzein	2.99 ± 0.09^{d}	3.14 ± 0.08^{c}	3.22 ± 0.10^{c}	3.46 ± 0.14^{b}	3.01 ± 0.09^{d}	3.38 ± 0.11^{b}	3.85 ± 0.10^{a}
Genistein	4.97 ± 0.32^{f}	6.93 ± 0.20^{d}	7.68 ± 0.40^{bc}	7.93 ± 0.27^{b}	5.35 ± 0.41^{e}	7.49 ± 0.25^{c}	8.58 ± 0.22^{a}
Glycitein	3.13 ± 0.14^{d}	3.39 ± 0.14^{c}	3.53 ± 0.21^{c}	3.76 ± 0.15^{b}	3.40 ± 0.16^{c}	3.80 ± 0.15^{b}	4.03 ± 0.14^{a}
Daidzin	57.47 ± 5.60^{f}	77.94 ± 4.55^{e}	106.56 ± 12.97^{cd}	116.22 ± 9.97^{c}	105.00 ± 7.25^{d}	170.72 ± 14.98^{a}	159.22 ± 13.73^{b}
Genistin	41.83 ± 4.68^{f}	63.22 ± 4.51^{e}	72.33 ± 4.71^{d}	78.72 ± 3.68^{c}	65.22 ± 5.78^{e}	103.39 ± 4.49^{b}	108.06 ± 2.26^{a}
Glycitin	< LOD	2.67 ± 0.17^{b}	2.55 ± 0.21^{b}	2.47 ± 0.25^{b}	2.71 ± 0.44^{b}	3.06 ± 0.19^{a}	3.18 ± 0.45^{a}
Acetyl Daidzin	54.33 ± 3.45^{f}	70.44 ± 2.89^{d}	72.78 ± 3.71^{d}	77.00 ± 2.06^{c}	66.72 ± 2.18^{e}	95.89 ± 1.95^{a}	91.00 ± 2.55^{b}
Acetyl Genistin	61.00 ± 2.84^{e}	81.17 ± 2.51^{d}	83.17 ± 2.54^{d}	89.72 ± 3.95^{c}	81.61 ± 5.25^{d}	111.28 ± 2.43^{a}	103.78 ± 5.19^{b}
Acetyl Glycitin	5.86 ± 0.24^{d}	6.72 ± 0.24^{b}	6.83 ± 0.36^{b}	6.06 ± 0.33^{d}	6.71 ± 0.31^{b}	7.67 ± 0.26^{a}	6.41 ± 0.19^{c}
Malonyl Daidzin	< LOD	< LOD	< LOD	< LOD	< LOD	< LOD	< LOD

Malonyl Genistin	< LOD	< LOD	< LOD	< LOD	< LOD	< LOD	< LOD
Malonyl Glycitin	ND	ND	ND	ND	ND	ND	ND
Total phenolic acids	25.88 ± 1.29^{d}	33.64 ± 2.57^{bc}	32.54 ± 2.67^{c}	36.20 ± 4.01^{b}	31.89 ± 3.34^{c}	39.25 ± 2.48^{a}	39.74 ± 2.77^{a}
Total isoflavones	231.58 ± 13.74^{g}	315.63 ± 9.71^{f}	358.64 ± 17.14^{d}	385.34 ± 13.93^{c}	339.74 ± 16.75^{e}	506.68 ± 18.17^{a}	488.11 ± 17.60^{b}
Sum of phenolic acids and isoflavones	257.47 ± 14.42^{g}	349.27 ± 10.38^{f}	391.18 ± 18.90^{d}	421.54 ± 14.10^{c}	371.63 ± 18.45^{e}	545.93 ± 18.03^{a}	527.85 ± 18.47^{b}

The values presented are the mean ± SEM. The superscript in each mean represents significant P values ($P < 0.05$) that are different from each other.

ND: not detected, < LOD: below the limit of detection.

Table S2. Change of composition and content of phenolic compounds in roasted soybean flour stored at high temperature (μg/g on dry weight basis).

	Storage duration						
	Initial	1 week	2 weeks	4 weeks	12 weeks	24 weeks	48 weeks
Protocatechuic acid	0.20 ± 0.01^{e}	0.24 ± 0.02^{c}	0.25 ± 0.01^{c}	0.22 ± 0.02^{d}	0.25 ± 0.02^{c}	0.47 ± 0.02^{b}	0.72 ± 0.03^{a}
p-Hydroxybenzoic acid	0.72 ± 0.14^{e}	1.85 ± 0.22^{d}	1.95 ± 0.28^{d}	2.05 ± 0.29^{d}	2.88 ± 0.33^{c}	14.11 ± 1.01^{b}	17.24 ± 1.55^{a}
Syringic acid	22.40 ± 1.06^{c}	29.84 ± 3.74^{a}	30.14 ± 2.38^{a}	30.29 ± 3.94^{a}	27.96 ± 1.56^{ab}	26.66 ± 1.20^{b}	22.58 ± 2.69^{c}
Salicylic acid	0.13 ± 0.01d^{e}	0.13 ± 0.01^{d}	0.12 ± 0.01^{e}	0.10 ± 0.00^{f}	0.15 ± 0.00^{c}	0.21 ± 0.01^{b}	0.27 ± 0.01^{a}
β-Resorcylic acid	0.12 ± 0.04^{d}	0.17 ± 0.01^{c}	0.18 ± 0.02^{bc}	0.13 ± 0.02^{d}	0.16 ± 0.01^{c}	0.20 ± 0.02^{b}	0.22 ± 0.02^{a}
p-Coumaric acid	0.96 ± 0.09^{b}	0.97 ± 0.08^{b}	0.95 ± 0.05^{b}	1.10 ± 0.07^{a}	0.93 ± 0.06^{b}	0.95 ± 0.05^{b}	1.06 ± 0.08^{a}
Chlorogenic acid	0.13 ± 0.01^{c}	0.13 ± 0.02^{bc}	0.13 ± 0.03^{bc}	0.15 ± 0.01^{ab}	0.10 ± 0.01^{d}	0.16 ± 0.01^{a}	0.13 ± 0.03^{bc}
Ferulic acid	1.24 ± 0.14^{c}	1.39 ± 0.09^{b}	1.42 ± 0.13^{ab}	1.51 ± 0.10^{a}	1.33 ± 0.10^{bc}	1.02 ± 0.09^{d}	0.88 ± 0.08^{e}
Daidzein	2.99 ± 0.09^{f}	3.15 ± 0.10^{de}	3.23 ± 0.09^{cd}	3.42 ± 0.13^{b}	3.13 ± 0.04^{e}	3.27 ± 0.07^{c}	3.67 ± 0.08^{a}
Genistein	4.97 ± 0.32^{e}	7.14 ± 0.29^{c}	7.68 ± 0.18^{b}	7.96 ± 0.16^{a}	6.32 ± 0.31^{d}	7.14 ± 0.17^{c}	7.87 ± 0.25^{ab}
Glycitein	3.13 ± 0.14^{c}	3.57 ± 0.10^{ab}	3.66 ± 0.15^{a}	3.61 ± 0.15^{a}	3.47 ± 0.10^{b}	3.00 ± 0.11^{d}	3.14 ± 0.10^{c}
Daidzin	57.47 ± 5.60^{f}	88.00 ± 7.61^{e}	119.00 ± 8.56^{b}	114.28 ± 8.87^{bc}	106.39 ± 6.43^{d}	128.17 ± 6.12^{a}	109.56 ± 12.09^{cd}
Genistin	41.83 ± 4.68^{d}	67.72 ± 4.06^{b}	75.78 ± 2.06^{a}	76.89 ± 4.98^{a}	69.94 ± 3.26^{b}	61.22 ± 3.29^{c}	40.06 ± 2.40^{d}
Glycitin	< LOD	2.80 ± 0.30^{ab}	2.88 ± 0.25^{ab}	2.62 ± 0.39^{bc}	2.76 ± 0.32^{abc}	2.92 ± 0.21^{a}	2.49 ± 0.25^{c}
Acetyl Daidzin	54.33 ± 3.45^{c}	71.28 ± 1.79^{b}	78.44 ± 8.03^{a}	77.56 ± 3.02^{a}	69.44 ± 2.16^{b}	79.22 ± 1.94^{a}	69.72 ± 2.08^{b}
Acetyl Genistin	61.00 ± 2.84^{d}	80.22 ± 1.50^{c}	87.22 ± 3.08^{a}	87.89 ± 3.05^{a}	83.94 ± 3.10^{b}	80.72 ± 1.68^{c}	79.00 ± 2.32^{c}
Acetyl Glycitin	5.86 ± 0.24^{c}	6.69 ± 0.33^{b}	7.34 ± 1.10^{a}	5.74 ± 0.27^{c}	6.59 ± 0.13^{b}	6.59 ± 0.17^{b}	5.61 ± 0.22^{c}
Malonyl Daidzin	< LOD	< LOD	< LOD	< LOD	< LOD	ND	ND

Malonyl Genistin	< LOD	< LOD	< LOD	< LOD	< LOD	< LOD	< LOD
Malonyl Glycitin	ND	ND	ND	ND	ND	ND	ND
Total phenolic acids	25.88 ± 1.29^{c}	34.73 ± 3.84^{b}	35.14 ± 2.34^{b}	35.55 ± 4.00^{b}	33.76 ± 1.70^{b}	43.76 ± 2.08^{a}	43.09 ± 3.73^{a}
Total isoflavones	231.58 ± 13.74^{e}	330.57 ± 8.83^{d}	385.23 ± 15.40^{a}	379.96 ± 14.28^{ab}	352.01 ± 8.45^{c}	372.26 ± 8.00^{b}	321.12 ± 10.41^{d}
Sum of phenolic acids and isoflavones	257.47 ± 14.42^{d}	365.30 ± 10.97^{c}	420.37 ± 15.33^{a}	415.51 ± 13.90^{a}	385.77 ± 8.70^{b}	416.02 ± 7.54^{a}	364.21 ± 12.65^{c}

The values presented are the mean ± SEM. The superscript in each mean represents significant P values ($P < 0.05$) that are different from each other.

ND: not detected, < LOD: below the limit of detection.

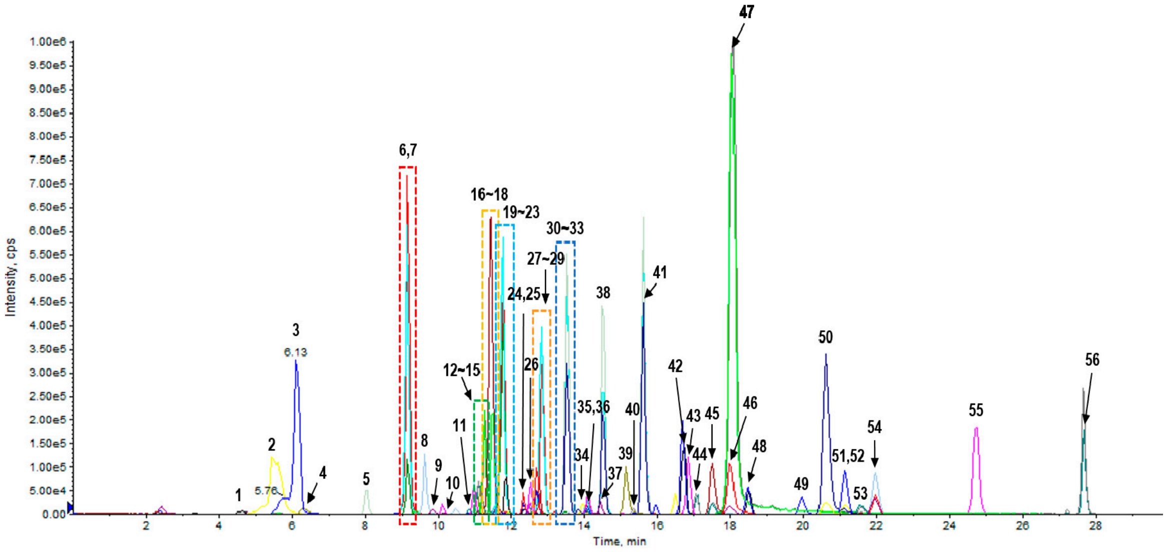

Figure S1. MRM ion chromatogram of standards of the 56 selected phenolic compounds and free amino acids.

1. L-Tyrosine; 2. 5-Sulfosalicylic acid; 3. Gallic acid; 4. L-Phenylalanine; 5. Homogentisic acid; 6. Protocatechuic acid; 7. Peonidin 3-*O*-glucoside chloride; 8. Chlorogenic acid; 9. Delphinidin chloride; 10. Catechin; 11. Cyanidin chloride; 12. Daidzin; 13. Glycitin; 14. Orientin; 15. *p*-Hydroxybenzoic acid; 16. Caffeic acid; 17. Rutin; 18. Syringic acid; 19. Gentisic acid; 20. Vitexin; 21. Pelargonidin chloride; 22. Malvidin chloride; 23. Peonidin chloride; 24. Polydatin; 25. Malonyl Glycitin; 26. Malonyl Daidzin; 27. Genistin; 28. Naringin; 29. *β*-Resorcylic acid; 30. Acetyl Daidzin; 31. *p*-Coumaric acid; 32. Acetyl Glycitin; 33. Vanillic acid; 34. Ferulic acid; 35. Malonyl Genistin; 36. Vanillin; 37. Veratric acid; 38. *m*-Coumaric acid; 39. Myricetin; 40. Acetyl Genistin; 41. *o*-Coumaric acid; 42. *trans*-Resveratrol; 43. Daidzein; 44. Glycitein; 45. Luteolin; 46. Quercetin; 47. Salicylic acid; 48. *cis*-Resveratrol; 49. *trans*-Cinnamic acid; 50. Apigenin; 51. Naringenin; 52. Genistein; 53. Kaempferol; 54. Hesperetin; 55. Formononetin; 56. Biochanin A.

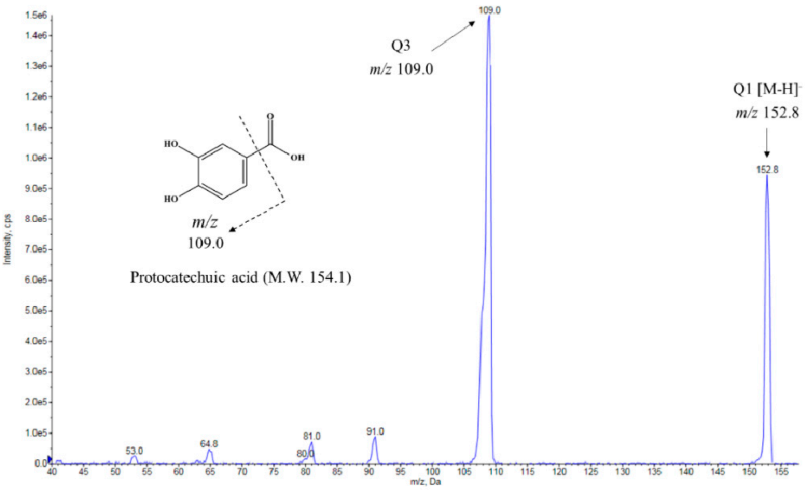
Q3
m/z 109.0
Q1 [M-H]⁻
m/z 152.8
m/z
109.0
Protocatechuic acid (M.W. 154.1)
Intensity, cps
m/z, Da

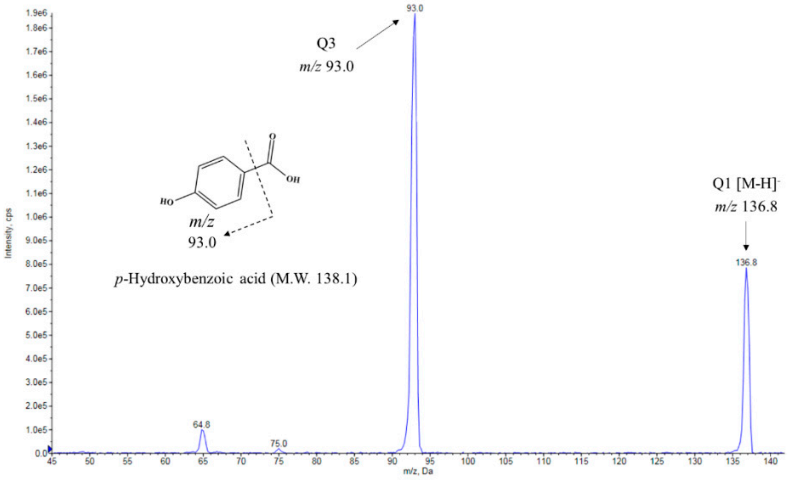
Q3
m/z 93.0
Q1 [M-H]⁻
m/z 136.8
m/z
93.0
p-Hydroxybenzoic acid (M.W. 138.1)
Intensity, cps
m/z, Da

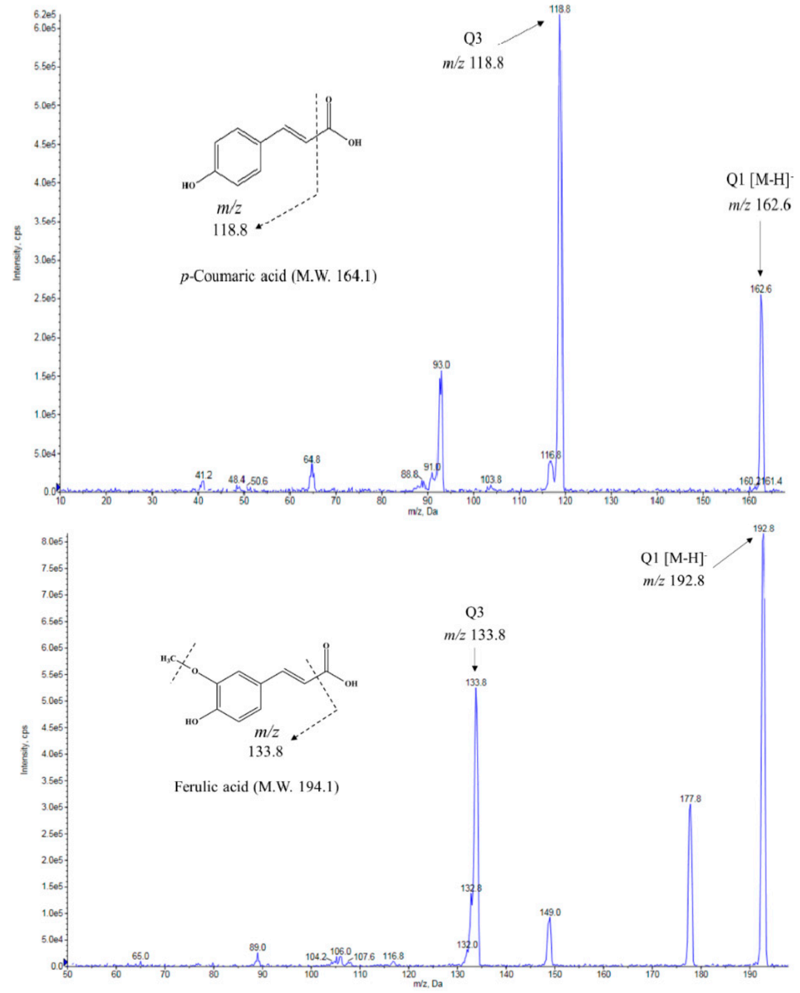

Figure S2. MS/MS spectra and fragmentation schemes of the representative phenolic acids.

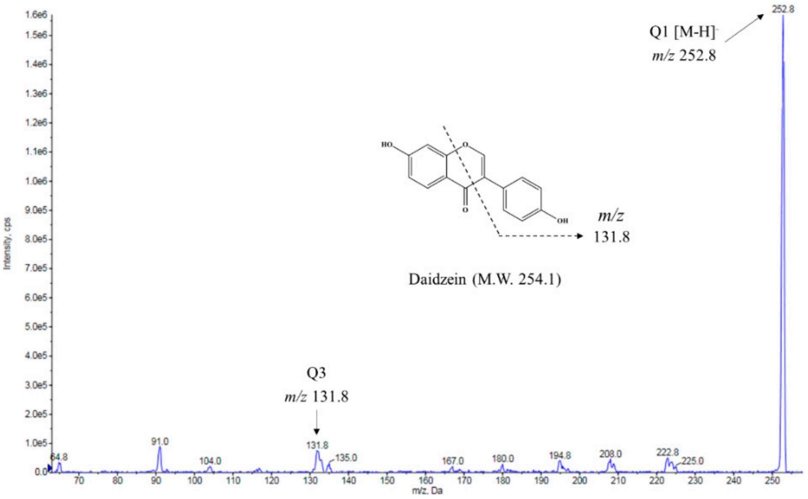
Q1 [M-H]⁻
m/z 252.8
m/z
131.8
Daidzein (M.W. 254.1)
Q3
m/z 131.8
Intensity, cps
m/z, Da

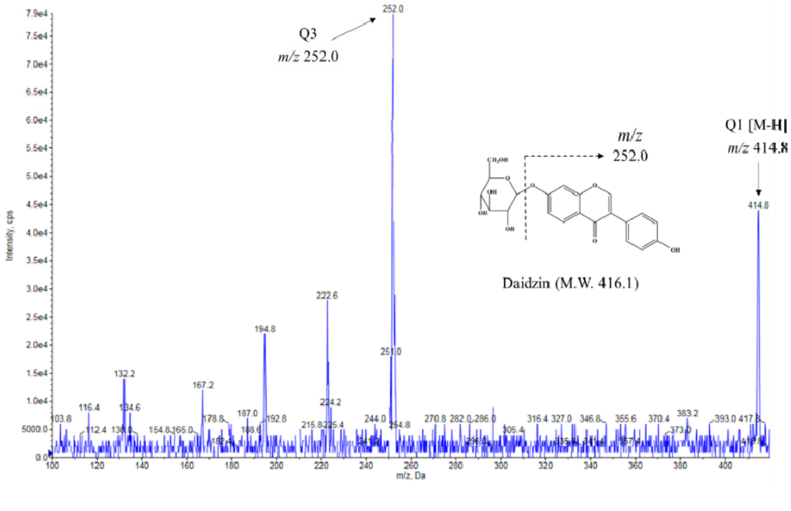
Q3
m/z 252.0
m/z
252.0
Q1 [M-H]
m/z 414.8
Daidzin (M.W. 416.1)
Intensity, cps
m/z, Da

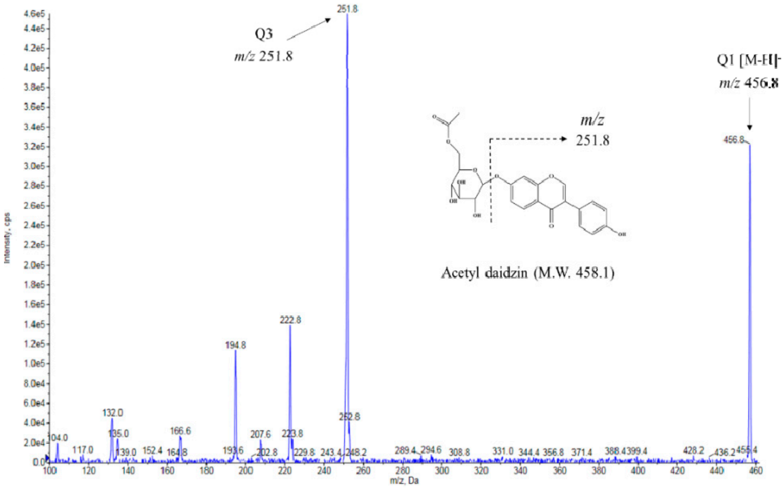

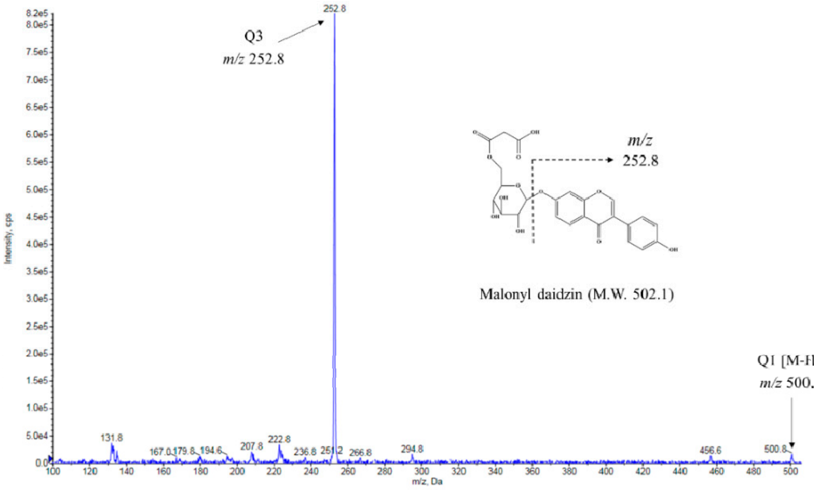

Figure S3. MS/MS spectra and fragmentation schemes of the representative isoflavone.
